# Supplementary material for: Transcriptional network involving ERG and AR orchestrates Distal-less homeobox-1 mediated prostate cancer progression
Source: Nat Commun. 2021 Sep 7;12:5325. doi: 10.1038/s41467-021-25623-2 (PMC8423767; doi:10.1038/s41467-021-25623-2)
Supplement: Supplementary file 1 — Supplementary Information [file 41467_2021_25623_MOESM1_ESM.pdf]

Supplementary Information

Transcriptional network involving ERG and AR orchestrates Distal-Less Homeobox-1 mediated prostate cancer progression

Sakshi Goel[1], Vipul Bhatia[1], Sushmita Kundu[1], Tanay Biswas[1], Shannon Carskadon[2], Nilesh Gupta[3], Mohammad Asim[4], Colm Morrissey[5], Nallasivam Palanisamy[2], Bushra Ateeq[1,6]*

[1]Molecular Oncology Laboratory, Department of Biological Sciences and Bioengineering, Indian Institute of Technology Kanpur, Kanpur, U.P., 208016, INDIA
[2]Vattikuti Urology Institute, Department of Urology, Henry Ford Health System, Detroit, MI 48202, USA
[3]Department of Pathology, Henry Ford Health System, Detroit, MI 48202, USA
[4]Department of Clinical and Experimental Medicine, Faculty of Health and Medical Sciences, University of Surrey, Guildford, UK
[5]Department of Urology, University of Washington, Seattle, WA 98195, USA
[6]The Mehta Family Center for Engineering in Medicine, Indian Institute of Technology Kanpur, Kanpur, U.P., 208016, INDIA

Running title: ERG/AR drives DLX1-mediated prostate cancer

Keywords: Prostate cancer; DLX1; ERG; AR; BET inhibitor

Financial support: This work is supported by the Science and Engineering Research Board (SERB) (EMR/2016/005273 to BA) and in part by the Wellcome Trust/ DBT India Alliance (IA/S/19/2/504659 to BA).

***Corresponding Author and Lead Contact**:
Dr. Bushra Ateeq
Molecular Oncology Laboratory,
Department of Biological Sciences and Bioengineering,
The Mehta Family Center for Engineering in Medicine,
Indian Institute of Technology Kanpur,
Kanpur, 208016, India
Phone: +91 512 2594083
Fax: +91 512 2594010
Email: bushra@iitk.ac.in (B. Ateeq)

Supplementary Figure 1

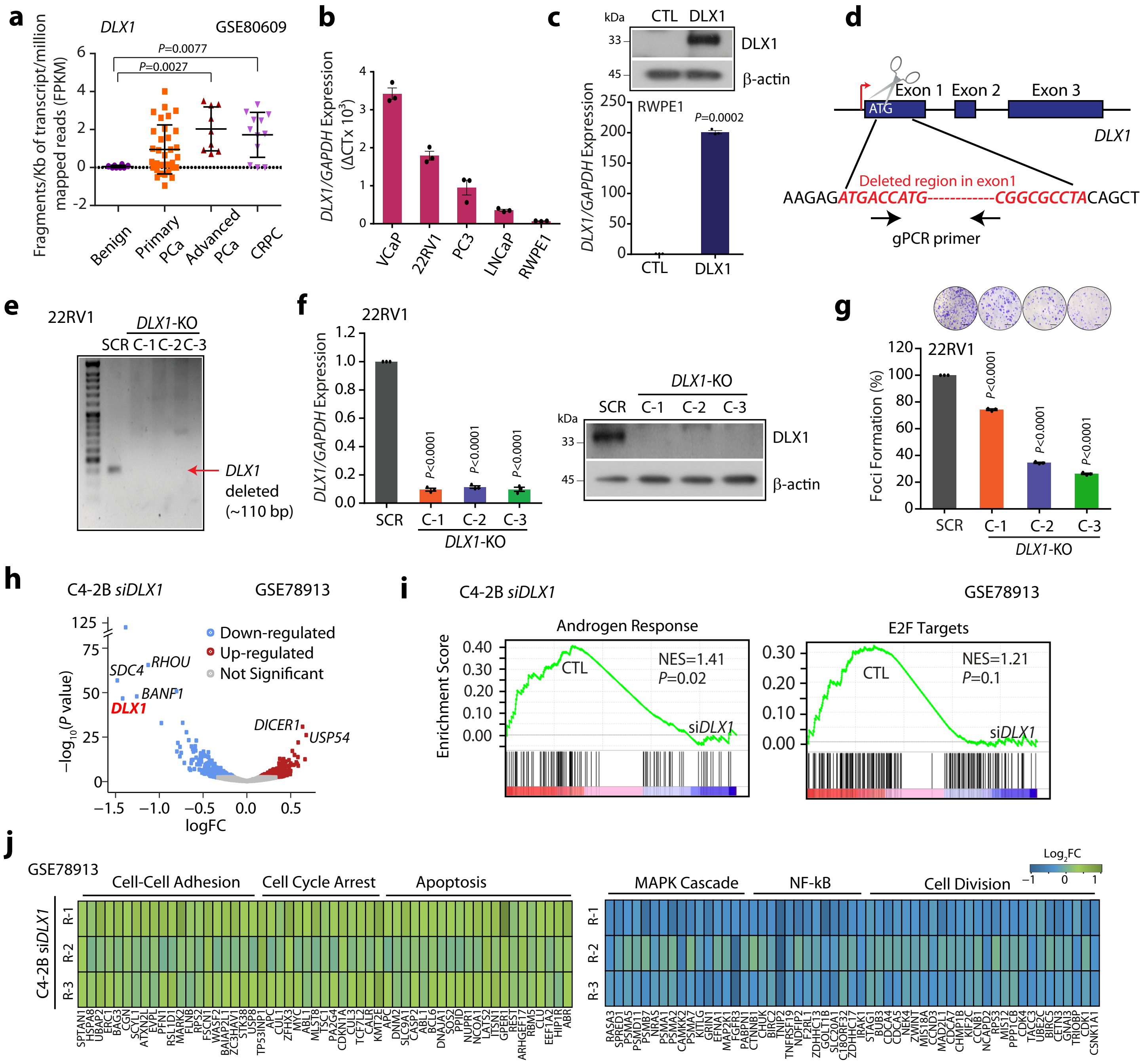

Supplementary Fig. 1. Characterization of DLX1 mediated oncogenicity and its associated biological pathways. **a** Dot-plot represents *DLX1* expression in RNA-Seq dataset (GSE80609) comprising of benign (n=8), primary PCa (n=32), advanced PCa (n=9) and CRPC (n=12) patient specimens, data represents FPKM and whiskers (error bars) denotes standard deviations (SD). **b** Q-PCR data showing *DLX1* expression in a panel of PCa cell lines. **c** Immunoblot (top panel) and Q-PCR (bottom panel) data showing relative expression of DLX1 in isogenic RWPE1 cells overexpressing DLX1 (*P*= 0.0002). **d** Schematic representation of *DLX1* genomic deletion to generate 22RV1-*DLX1*-KO cells. **e** Agarose gel depicting deleted region of *DLX1* in KO clones and control SCR cells. **f** Q-PCR (left panel) and immunoblot analysis (right panel) showing DLX1 expression in 22RV1-*DLX1*-KO cells (*P*< 0.0001). **g** Foci formation assay using same cells as **f** (*P*< 0.0001). Representative images are shown as inset. Scale bar 1000μm. **h** Volcano plot showing deregulated genes upon silencing *DLX1* in C4-2B cells using RNA-Seq dataset (GSE78913). Wald test statistics is used to identify *P* value and is further adjusted using Benjamini-Hochberg multiple test correction. **i** Same as **h**, except GSEA plots showing deregulated pathways. **j** Same as **h**, except heatmaps displaying deregulated genes involved in biological processes supporting oncogenesis. R-1, R-2 and R-3 represents three independent replicates.

Data shown from three biological independent samples (n=3). Data represents mean ± SEM or otherwise specified. For panels, **a, f** and **g** One-way ANOVA, Dunnett's multiple comparison test, **c** Unpaired t-test with Welch's correction was applied. Source data are provided as a Source Data file.

Supplementary Figure 2

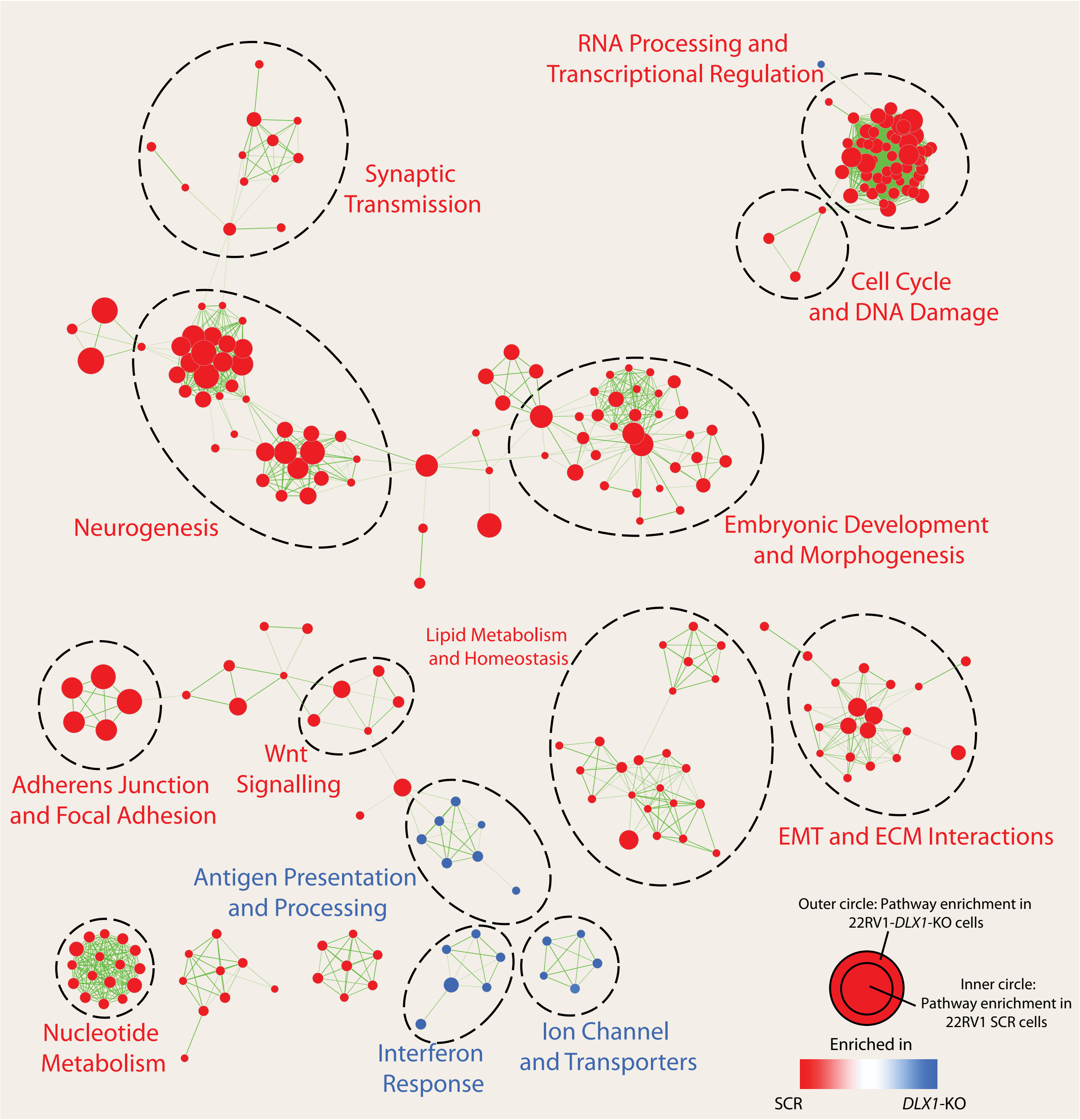

Supplementary Fig. 2. Enrichment map showing overlapping biological networks. GO term enrichment map for the gene expression profiles obtained from microarray analysis of 22RV1-*DLX1*-KO and control SCR cells. Each node represents a GO term and the size of the node corresponds to the number of genes involved in a particular GO term. Red dots indicate the pathways enriched in control group while blue dots depict pathways enriched in *DLX1*-KO cells. Intensity of the colours represent the enrichment significance. Each cluster is manually labelled to depict the pathway enrichment.

Supplementary Figure 3

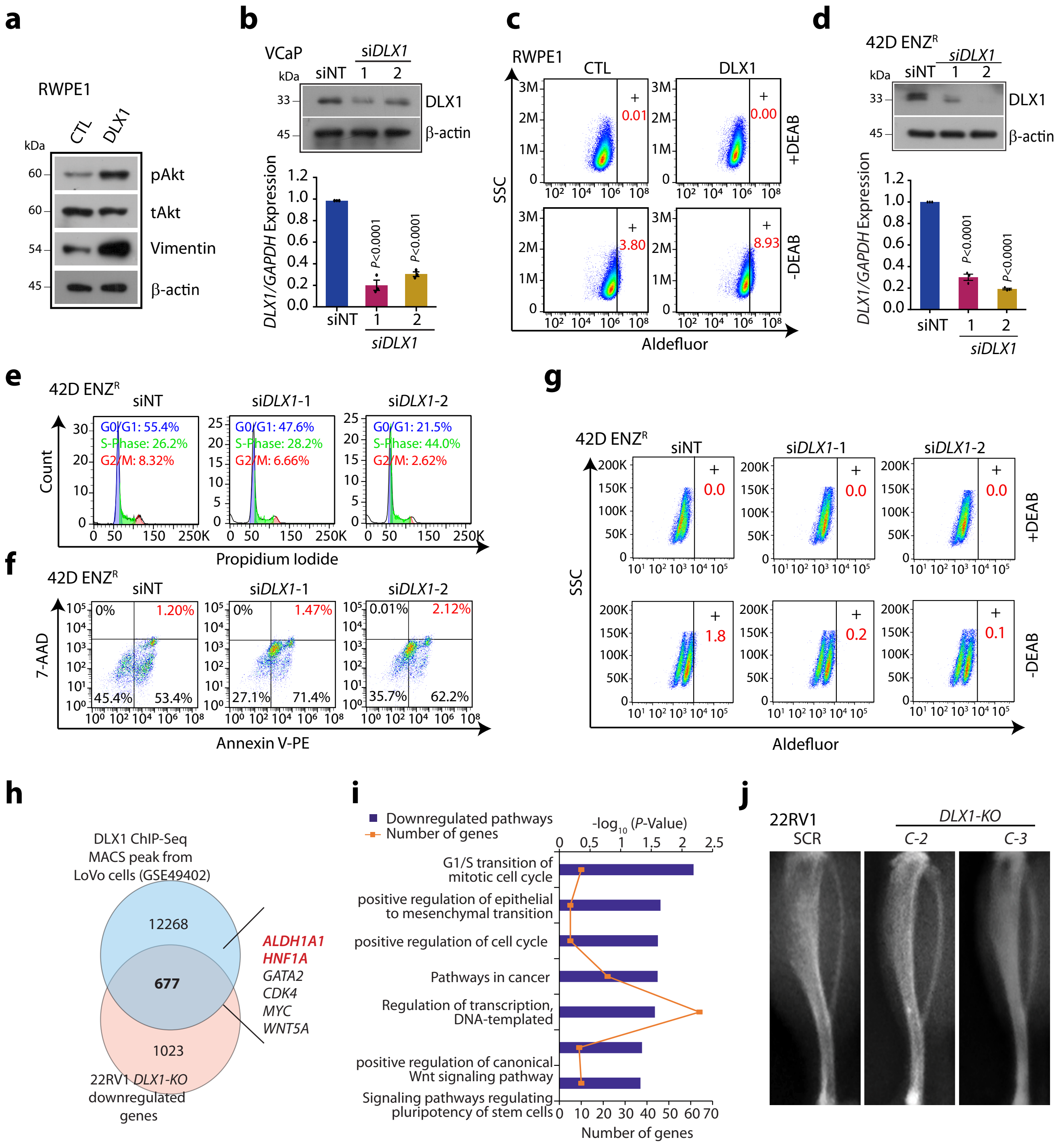

Supplementary Fig. 3. DLX1 regulates oncogenic pathways involved in prostate cancer progression and metastases. a Immunoblot showing expression of phospho (p) and total (t) Akt and vimentin in isogenic RWPE1 cells overexpressing DLX1. β-actin is used as a positive control. **b** Immunoblot (top panel) and Q-PCR (bottom panel) data showing relative expression of *DLX1* in siRNA-mediated *DLX1*-silenced VCaP cells ($P < 0.0001$). **c** Fluorescence intensity of catalyzed ALDH substrate using same cells as **a**. Marked windows show ALDH1+ percent cell population. **d** Immunoblot (top panel) and Q-PCR (bottom panel) data showing relative expression of DLX1 in siRNA-mediated *DLX1*-silenced 42D ENZ^R cells ($P < 0.0001$). **e** Flow cytometry data for cell cycle distribution of cells same cells as in **d**. **f** Apoptosis assay using same cells as in **d**. **g** Fluorescence intensity of catalyzed ALDH substrate using cells as same in **d**. Marked windows show ALDH1+ percent cell population. **h** Venn diagram showing common downregulated genes in 22RV1-*DLX1*-KO cells and DLX1 ChIP-Seq data (GSE49402) for LoVo cells. **i** DAVID analysis showing down-regulated biological processes associated with common genes identified in **h**. **j** Representative images of the X-ray scans of mice tibia injected with 22RV1-SCR and *DLX1*-KO cells.
Data shown from three biological independent samples (n=3). Data represents mean ± SEM. For panels, **b** and **d** one-way ANOVA, Dunnett's multiple comparison test was applied. Source data are provided as a Source Data file.

Supplementary Figure 4

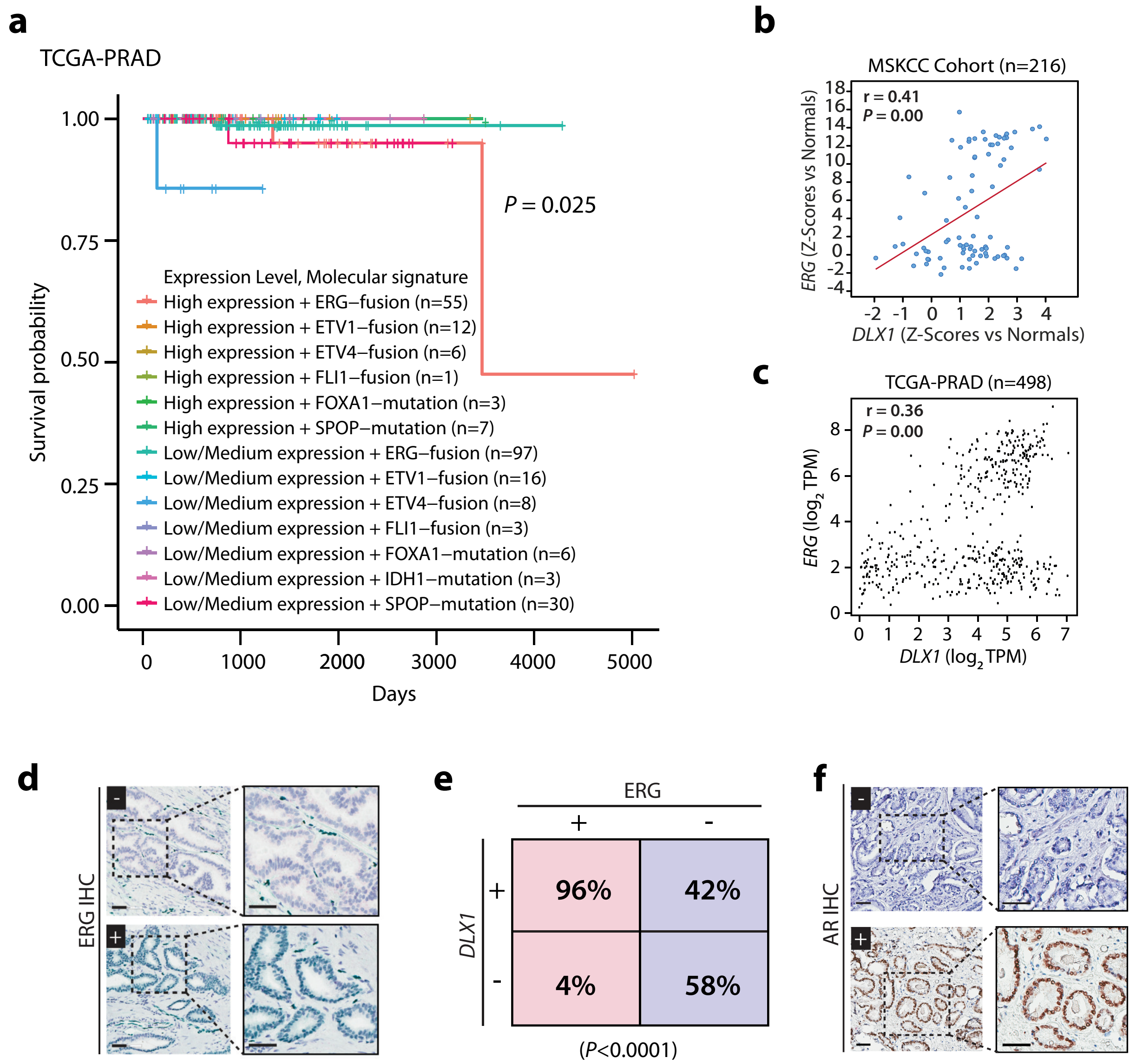

Supplementary Fig. 4. DLX1 and ERG shows positive association in PCa patients. a Kaplan-Meier plot depicting survival probability considering the level of *DLX1* expression in association of molecular subtypes in primary prostate cancer patients accessible from the TCGA-PRAD dataset. **b** Correlation plot between *DLX1* and *ERG* using Memorial Sloan Kettering Cancer Center cohort (MSKCC) dataset available at the cBioPortal. Two-sided t-test was applied to calculate *P*-value. **c** Correlation plot between *DLX1* and *ERG* transcript per million read count (TPM) in TCGA-PRAD dataset generated using GEPIA (Gene Expression Profiling Interactive Analysis). Two-sided t-test was applied to calculate *P*-value. **d** Representative core of PCa TMA cores (n=144) immunostained for ERG. Scale bar, 50µm. **e** Contingency table depicting status of *DLX1* and ERG. Pink panel shows status of DLX1 patients in ERG positive cases (left) and blue panel shows status of *DLX1* in ERG negative cases (right). *P*-value for Fisher's exact test is indicated. **f** same as in **d,** except for IHC staining for AR. Scale bar, 50µm. Source data for panel **e** is provided as a Source Data file.

Supplementary Figure 5

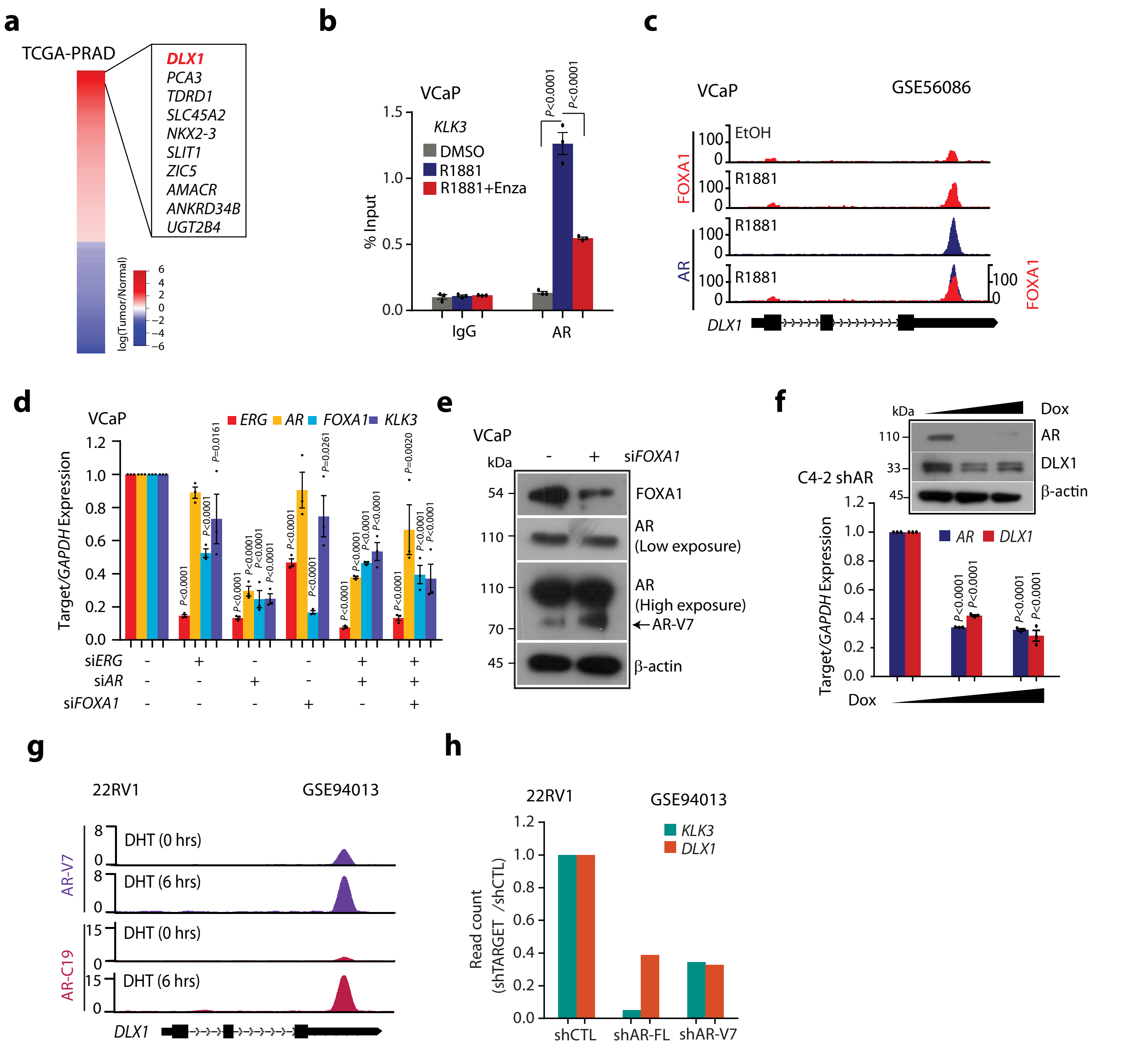

Supplementary Fig. 5. AR gets recruited at DLX1 putative enhancer in PCa cells. a Heatmap showing top ten differentially upregulated genes with AR binding sites within 50kb nearby region from TCGA-PRAD dataset. **b** ChIP-qPCR data showing AR recruitment at the *KLK3* promoter in VCaP cells treated with vehicle control, R1881 or a combination of R1881 and Enza (P< 0.0001). **c** ChIP-Seq data depicting recruitment of AR and FOXA1 at the same genomic loci of *DLX1* gene in R1881-stimulated VCaP cells. **d** Q-PCR data depicting relative expression of target genes in VCaP cells transfected with siRNA against *AR*, *ERG* and/or *FOXA1*. **e** Immunoblot for FOXA1 and AR in *FOXA1*-silenced VCaP cells. β-actin is used as a loading control. **f** Immunoblot (top panel) and Q-PCR (bottom panel) data for AR and DLX1 in doxycycline (dox) inducible shRNA against *AR* in C4-2 cells treated with dox for 48 hours (P< 0.0001). **g** ChIP-Seq data (GSE94013) using AR-V7 and AR C-terminal (AR-C19) specific antibodies in DHT stimulated 22RV1 cells. **h** Bar plot showing relative read count of *KLK3* and *DLX1* obtained from RNA-Seq data (GSE94013) for *AR-FL* and *AR-V7* knockdown in 22RV1 cells.
Data shown from three biological independent samples (n=3). Data represents mean ± SEM. For panels **b** and **d** two-way ANOVA Tukey's multiple comparison test; **f** two-way ANOVA Sidak's multiple comparison test was applied. Source data are provided as a Source Data file.

Supplementary Figure 6

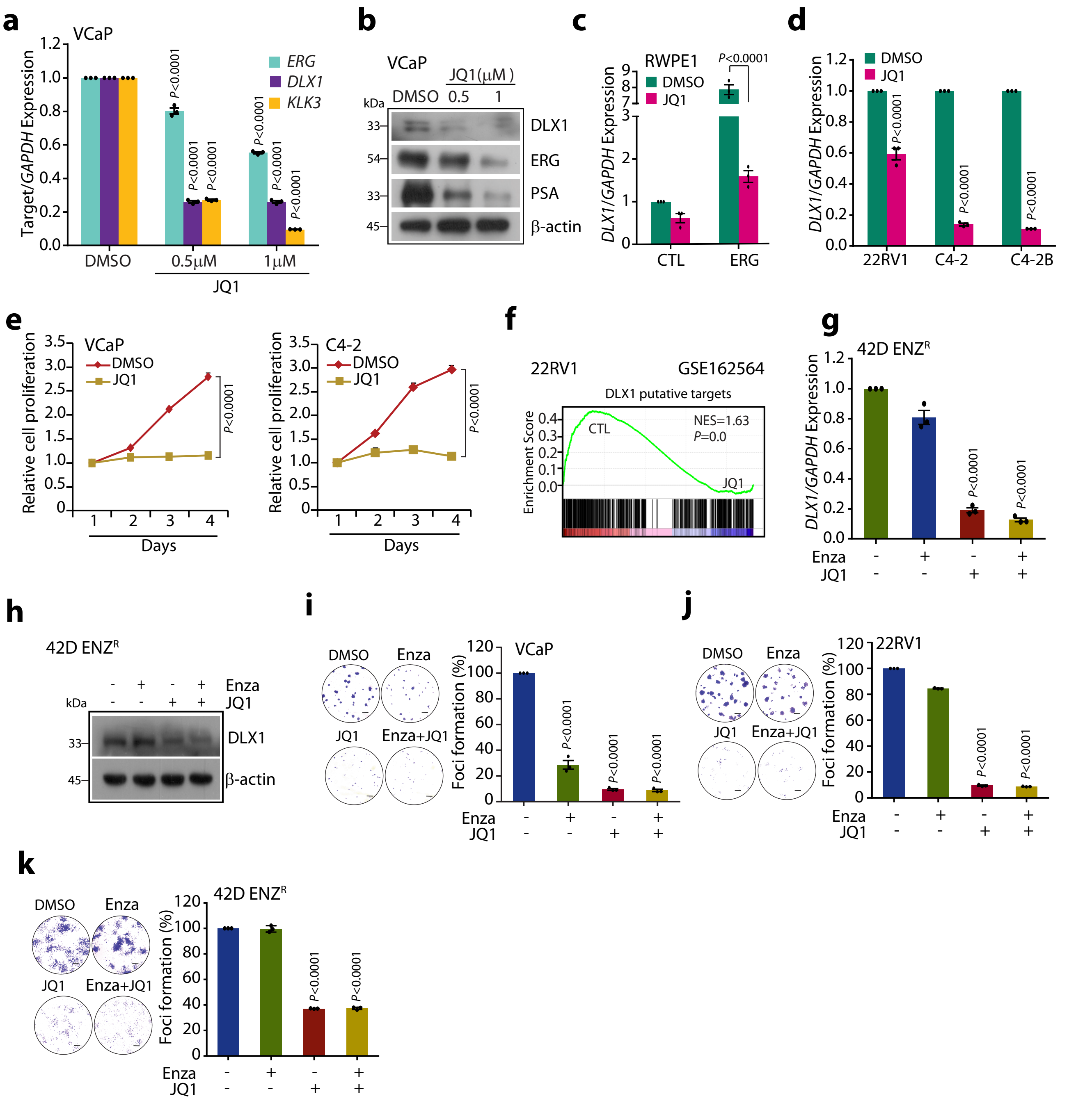

Supplementary Fig. 6. BET inhibitor attenuates DLX1 expression and its mediated oncogenic properties. a Q-PCR data showing relative expression of target genes in JQ1 treated VCaP cells. *KLK3* used as a positive control (P< 0.0001). **b** Same as **a,** except immunoblot data. β-actin was used as positive control. **c** Q-PCR data showing *DLX1* in RWPE1-ERG and control cells treated with JQ1 (0.5µM) (P< 0.0001). **d** Q-PCR data showing *DLX1* expression in 22RV1, C4-2 and C4-2B cells treated with JQ1 (0.5µM) (P< 0.0001). **e** Cell proliferation assay using VCaP and C4-2 cells upon JQ1 treatment at the indicated time points (P< 0.0001). **f** GSEA plot showing DLX1 putative target genes in RNA-Seq data (GSE162564) of JQ1 treated 22RV1 cells. **g** Q-PCR data showing *DLX1* expression in 42D ENZ^R cells (P< 0.0001). **h** Same as **g,** except immunoblot. **i** Bar plot representing percent foci formed in VCaP cells treated with JQ1 and Enza (P< 0.0001). **j** Same as **i,** except for 22RV1 cells (P< 0.0001). **k** Same as **i,** except for 42D ENZ^R cells (P< 0.0001). Representative images for panels **i, j** and **k** are shown on the left of bar plot. Scale bar 500µm.

Data shown from three biological independent samples (n=3). Data represents mean ± SEM. For panels **a** two-way ANOVA Dunnett's multiple comparison test; **c** two-way ANOVA Tukey's multiple comparison test; **d** and **e** two-way ANOVA Sidak's multiple comparison test; **g, i, j** and **k** one-way ANOVA Tukey's multiple comparison test was applied. Source data are provided as a Source Data file.

Supplementary Figure 7

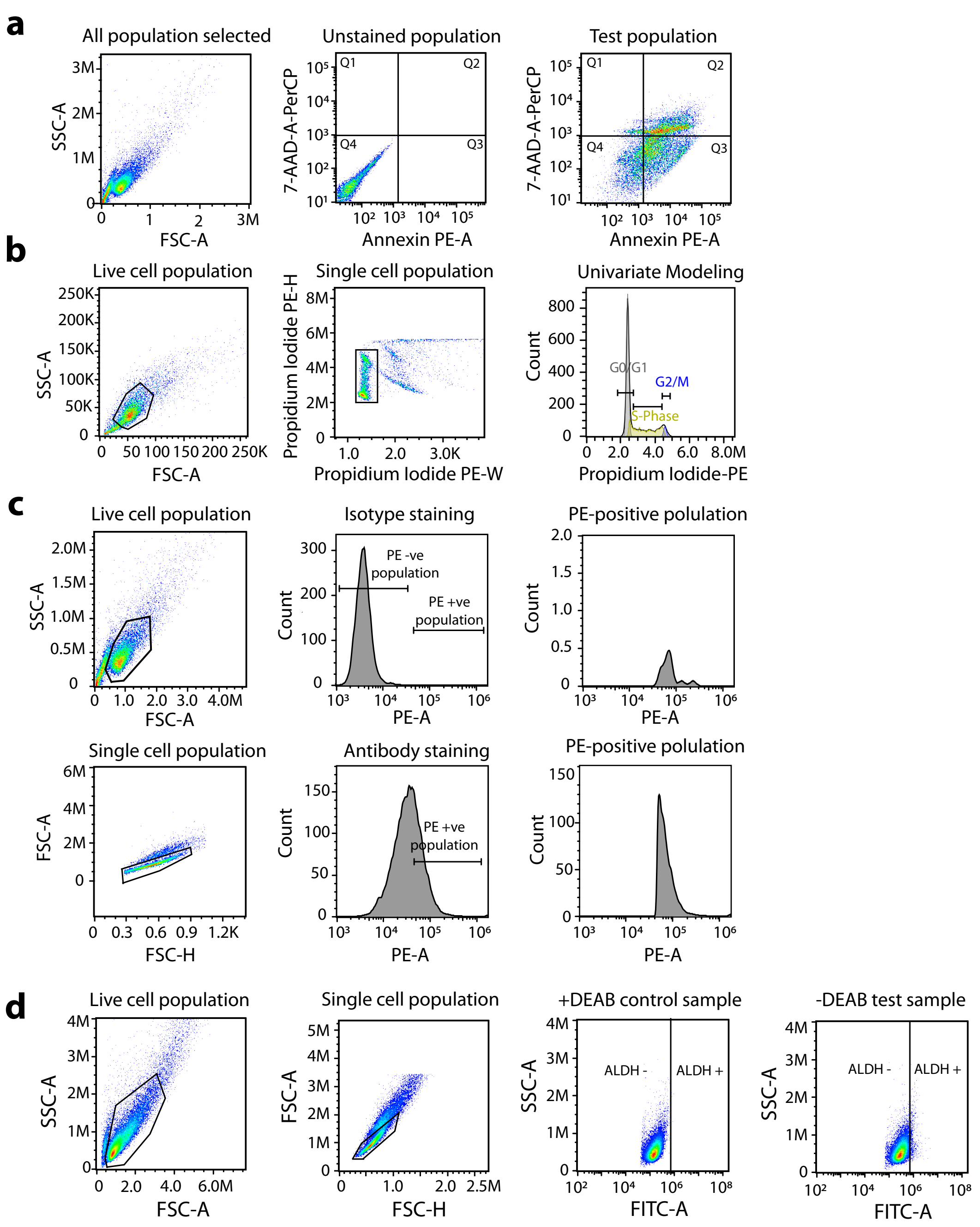

Supplementary Fig. 7. Gating strategy applied for the flow cytometry experiments. a Representative graph showing the gating strategy used for the apoptosis assay depicted in **Fig. 2d** and **supplementary Fig. 3f.** Quadrants were gated on the dot plots of Annexin (PE) versus 7AAD (PerCP) channels using unstained sample. The quadrants were defined as Q1: Annexin-/7AAD+ (+ positive) representing necrotic cells, Q2: Annexin+/7AAD+ showing late apoptotic cells, Q3: Annexin+/7AAD– (– negative) depicting early apoptotic cells, and Q4: Annexin–/7AAD– showing viable cell population. Same gate was applied on the experimental cell population to analyze apoptosis. **b** Dot plot representing the gating technique for the propidium iodide cell cycle analysis depicted in **Fig. 2e** and **supplementary Fig. 3e**. Live cell population was first gated using forward scatter (FSC) versus side scatter (SSC) channels, followed by identifying single cell population. In-built univariate model in the FlowJo software was used to determine the cell cycle distribution. **c** Representative graph showing gating strategy for the expression analysis of cell-surface markers shown in **Fig. 2g**. Gate was applied to identify viable-cell population using FSC versus SSC dot plot, followed by gating single cell population. Histograms were generated using isotype control to gate the positive cell population. **d** Dot plots representing gating strategy for ALDH activity assay depicted in **Fig. 2h, i, 7m-o** and **supplementary Fig. 3c and g**. Viable cell population was gated followed by gating single-cell population in FSC-H versus FSC-A channel dot plot. DEAB containing control sample tube was used to gate ALDH + population on the FITC versus SSC dot plot.

Supplementary Table 1: List of Primers used in this study

**Quantitative PCR (qPCR) primers**			
S.No.	Gene	Primer Name	
1	*DLX1*	qDLX1_FP	GCG GCC TCT TTG GGA CTC ACA C
		qDLX1_RP	GGC CAA CGC ACT ACC CTC CAG A
2	*CDH1*	qCDH1_FP	CTT CTG CTG ATC CTG TCT GAT G
		qCDH1_RP	TGC TGT GAA GGG AGA TGT ATT G
3	*VIMENTIN*	qVIM_FP	GAT TCA CTC CCT CTG GTT GAT AC
		qVIM_RP	GTC ATC GTG ATG CTG AGA AGT
4	*SNAIL*	qSNAIL_FP	CCT TCG TCC TTC TCC TCT ACT T
		qSNAIL_RP	TTC GAG CCT GGA GAT CCT T
5	*POU5F1/Oct-4*	qOCT_FP	GGA GGA AGC TGA CAA TGA AA
		qOCT_RP	GGC CTG CAC GAG GTT TT
6	*ABCG2*	qABCG2_FP	GTA AAG CAG GGC ATC GAT CT
		qABCG2_RP	CAG GTA GGC AAT TGT GAG GAA
7	*CD117(C-KIT)*	qKIT_FP	CAA GGC TTC TCC AAT TCT GC
		qKIT_RP	TGC AGT GGT CCA CAG AAG AG
8	*SOX2*	qSOX2_FP	CAT GGG TTC GGT GGT CAA G
		qSOX2_RP	TGA TCA TGT CCC GGA GGT
9	*ERG*	qERG_FP	CGC AGA GTT ATC GTG CCA GCA GAT
		qERG_RP	CCA TAT TCT TTC ACC GCC CAC TCC
10	*KLK3*	qPSA_FP	GTC TGC GGC GGT GTT CTG
		qPSA_RP	TGC CGA CCC AGC AAG ATC
11	*AR*	qAR_FP	AAT CCC ACA TCC TGC TCA AG
		qAR_RP	GAG TCC AGG AGC TTG GTG AG
12	*FOXA1*	qFOXA1_FP	ATA CTC GCC TTA CGG CTC TA
		qFOXA1_RP	GTT TAG GAC GGG TCT GGA ATA C
13	*ALDH1A1*	qALDH1A1_FP	GGC TTA TCA GCA GGA GTG TTT A
		qALDH1A1_RP	ACC ACG CCA TAG CAA TTC A
14	*HNF1A*	qHNF1A_FP	ACG AGA CGG ACG ACG AT
		qHNF1A_RP	CTT GCA GAA GGG TCT CCA C
15	*GATA2*	qGATA2_FP	GAC GAC AAC CAC CAC CTT AT
		qGATA2_RP	AGT CTG GAT CCC TTC CTT CT
16	*GAPDH*	qGAPDH_FP	TGC ACC ACC AAC TGC TTA GC
		qGAPDH_RP	GGC ATG GAC TGT GGT CAT GAG
**Chromatin Immunoprecipitation (ChIP-qPCR) primers**			
1	*KLK3*	KLK3_FP	CCT AGA TGA AGT CTC CAT GAG CTA CA
		KLK3_RP	GGG AGG GAG AGC TAG CAC TTG
2	*DLX1*	EBM1_FP	AGC TTT GAA CCG AGT TTG GG
		EBM1_RP	TTC TCT CCT CTG CTT CCC TTT
3	*DLX1*	EBM2_FP	CAG CCC ATT GTG CTT CCT G
		EBM2_RP	GGT CCG CTG TCT TGC ATA ATC
4	*DLX1*	ARE1_FP	CGC CTG GAC AAG AAG GAA A
		ARE1_RP	CCA CTG GAA TTA GAG TGC TTG T
5	*DLX1*	ARE2_FP	GAG AAA TGG ACT TCG CCT GA
		ARE2_FP	CCC GTG CGC TTA AAG TAA AC

6	*CUTL2*	CUTL2_FP	AAA CAT GTC TCC CCA TGG AA
		CUTL2_RP	GGT ACA TCC TGC ACC AGA CC
7	*ALDH1A1*	DBM1_FP	TGG TGA ACT AAA CCA CAC TGA T
		DBM1_RP	ACT GAG TGC CAT TTC ACA GG
8	*ALDH1A1*	DBM2_FP	ACT TTA GCC CGT GCA GAT AAA
		DBM2_RP	GAC ATT TCT GAT TCG GCT CCT
9	*HNF1A*	DBM1_FP	GTT TAT AAC TAA CTA CCT GGG TTA C
		DBM1_RP	GCC CAG GCC TTC AAT AG
10	*HNF1A*	DBM2_FP	GGA AAG AAG GGA GAG AGA GA
		DBM2_RP	CAC TTG TGC AAG GTG ATC TA
**Genomic PCR primers**			
1	*DLX1*	gDLX1del_FP	ATG ACC ATG ACC ACC ATG CCA
		gDLX1del_RP	AAT GGC CCG CCG AGT GTA AA
2	TaqMan-*Alu*	hAlu_FP	GTC AGG AGA TCG AGA CCA TCC T
		hAlu_RP	AGT GGC GCA ATC TCG GC
**Mutagenesis Primers**			
1	*DLX1*	MT1_FP	CCA TTG TGC AAC CTG CCC GG
		MT1_RP	CCG GGC AGG TTG CAC AAT GG
2	*DLX1*	MT2_FP	TTG GGT TCC AGC CTG TCC TGA
		MT2_RP	TCA GGA CAG GCT GGA ACC CAA
