# Supplementary material for: Transcriptional network involving ERG and AR orchestrates Distal-less homeobox-1 mediated prostate cancer progression
Source: Nat Commun. 2021 Sep 7;12:5325. doi: 10.1038/s41467-021-25623-2 (PMC8423767; doi:10.1038/s41467-021-25623-2)
Supplement: Supplementary file 3 — Description of Additional Supplementary Files [file 41467_2021_25623_MOESM3_ESM.pdf]

Description of Additional Supplementary Files

File Name: Supplementary Data 1

Description: Deregulated biological processes in 22RV1-*DLX1*-KO cells predicted by DAVID analysis.
