# Supplementary material for: Transcriptional network involving ERG and AR orchestrates Distal-less homeobox-1 mediated prostate cancer progression
Source: Nat Commun. 2021 Sep 7;12:5325. doi: 10.1038/s41467-021-25623-2 (PMC8423767; doi:10.1038/s41467-021-25623-2)
Supplement: Supplementary file 5 — Reporting Summary [file 41467_2021_25623_MOESM5_ESM.pdf]

nature portfolio

Corresponding author(s): Dr. Bushra Ateeq

Last updated by author(s): Aug 2, 2021

Reporting Summary

Nature Portfolio wishes to improve the reproducibility of the work that we publish. This form provides structure for consistency and transparency in reporting. For further information on Nature Portfolio policies, see our Editorial Policies and the Editorial Policy Checklist.

Statistics

For all statistical analyses, confirm that the following items are present in the figure legend, table legend, main text, or Methods section.

n/a	Confirmed	
☐	☒	The exact sample size (*n*) for each experimental group/condition, given as a discrete number and unit of measurement
☐	☒	A statement on whether measurements were taken from distinct samples or whether the same sample was measured repeatedly
☐	☒	The statistical test(s) used AND whether they are one- or two-sided<br>*Only common tests should be described solely by name; describe more complex techniques in the Methods section.*
☒	☐	A description of all covariates tested
☐	☒	A description of any assumptions or corrections, such as tests of normality and adjustment for multiple comparisons
☐	☒	A full description of the statistical parameters including central tendency (e.g. means) or other basic estimates (e.g. regression coefficient) AND variation (e.g. standard deviation) or associated estimates of uncertainty (e.g. confidence intervals)
☐	☒	For null hypothesis testing, the test statistic (e.g. *F*, *t*, *r*) with confidence intervals, effect sizes, degrees of freedom and *P* value noted<br>*Give P values as exact values whenever suitable.*
☒	☐	For Bayesian analysis, information on the choice of priors and Markov chain Monte Carlo settings
☒	☐	For hierarchical and complex designs, identification of the appropriate level for tests and full reporting of outcomes
☐	☒	Estimates of effect sizes (e.g. Cohen's *d*, Pearson's *r*), indicating how they were calculated

Our web collection on statistics for biologists contains articles on many of the points above.

Software and code

Policy information about availability of computer code

Data collection	Data were collected using Microsoft Excel Office 2016. TCGA-PRAD PCa patient data was collected from UCSC Xena online browser. MSKCC and SU2C PCa patient data was collected from cBioportal open source online browser.
Data analysis	Data was analyzed using Microsoft Excel Office 2016, GraphPad Prism version 7.0, Integrated Genome Browser 9.0.1, Integrated Genome Viewer, Galaxy, SAM tools, Bowtie 2, MACS, Trimmomatic, HISAT2, DESeq2, DAVID Bioinformatics Database, JASPAR, MatInspector, Gene Set Enrichment Analysis 4.0.1, FlowJo v10.6.1 and R 3.6.1., ImageJ 1.53c, Imagejs Ki-67 online module, GEPIA, UALCAN, CTAn v1.16.8.0 software.

For manuscripts utilizing custom algorithms or software that are central to the research but not yet described in published literature, software must be made available to editors and reviewers. We strongly encourage code deposition in a community repository (e.g. GitHub). See the Nature Portfolio guidelines for submitting code & software for further information.

Data

Policy information about availability of data

All manuscripts must include a data availability statement. This statement should provide the following information, where applicable:

- Accession codes, unique identifiers, or web links for publicly available datasets
- A description of any restrictions on data availability
- For clinical datasets or third party data, please ensure that the statement adheres to our policy

The gene expression microarray data from this study has been submitted to the NCBI Gene Expression Omnibus (GEO), under the accession number GSE138738 (https://www.ncbi.nlm.nih.gov/geo/query/acc.cgi?acc=GSE138738). Other datasets used in this study includes downloaded from GEO (www.ncbi.nlm.nih.gov/geo) includes: Gene expression profiling in PCa patients, GSE35988 (https://www.ncbi.nlm.nih.gov/geo/query/acc.cgi?acc=GSE35988) and GSE80609 (https://www.ncbi.nlm.nih.gov/geo/query/acc.cgi?acc=GSE80609); RNA-Seq data of RWPE1, GSE128399 (https://www.ncbi.nlm.nih.gov/geo/query/acc.cgi?acc=GSE128399)

22RV1 and VCaP cell, GSE118206 (https://www.ncbi.nlm.nih.gov/geo/query/acc.cgi?acc=GSE118206); RNA-Seq data od DLX1 silenced C4-2B cells, GSE78913 (https://www.ncbi.nlm.nih.gov/geo/query/acc.cgi?acc=GSE78913); ChIP-Seq data for ERG binding in VCaP cells, GSE98809 (https://www.ncbi.nlm.nih.gov/geo/query/acc.cgi?acc=GSE98809), in ERG silenced VCaP cells, GSE110655 (https://www.ncbi.nlm.nih.gov/geo/query/acc.cgi?acc=GSE110655), in RWPE1 cells, GSE37752 (https://www.ncbi.nlm.nih.gov/geo/query/acc.cgi?acc=GSE37752); ChIP-Seq data for AR and ERG binding in R1881 stimulated VCaP cells, GSE28951 (https://www.ncbi.nlm.nih.gov/geo/query/acc.cgi?acc=GSE28951); ChIP-Seq for AR binding in PCa patient samples, GSE70079 (https://www.ncbi.nlm.nih.gov/geo/query/acc.cgi?acc=GSE70079); ChIP-Seq in VCaP cells for ERG, AR, H3K27Ac, and RNA-PolII, GSE55062 (https://www.ncbi.nlm.nih.gov/geo/query/acc.cgi?acc=GSE55062), and FOXA1, GSE56086 (https://www.ncbi.nlm.nih.gov/geo/query/acc.cgi?acc=GSE56086); ChIA-PET for RNA-PolII in VCaP cells, GSE121020 (https://www.ncbi.nlm.nih.gov/geo/query/acc.cgi?acc=GSE121020); ChIP-Seq for ERG and H3K27Ac in JQ1 treated VCaP cells, GSE55064 (https://www.ncbi.nlm.nih.gov/geo/query/acc.cgi?acc=GSE55064); ChIP-Seq for DLX1 in LoVo colorectal cancer cells, GSE49402 (https://www.ncbi.nlm.nih.gov/geo/query/acc.cgi?acc=GSE49402); ChIP-Se1 for FOXA1 and AR in R1881 stimulated VCaP cells, GSE56086 (https://www.ncbi.nlm.nih.gov/geo/query/acc.cgi?acc=GSE56086); ChIP-Seq using AR-C19 and AR-V7 specific antibody in 22RV1 cells, GSE94013 (https://www.ncbi.nlm.nih.gov/geo/query/acc.cgi?acc=GSE94013); RNA-Seq data of 22RV1 cells, GSE94013; RNA-Seq data of JQ1 treated 22RV1 cells, GSE162564 (https://www.ncbi.nlm.nih.gov/geo/query/acc.cgi?acc=GSE162564). Human reference genome (hg19) was downloaded from UCSC genome browser (https://hgdownload.soe.ucsc.edu/downloads.html#human). There are various databases used in this study, namely: UCSC Xena (https://xenabrowser.net/) to download TCGA-PRAD dataset, cBioPortal (https://www.cbioportal.org/) to download MSKCC correlation plot and SU2C dataset (https://www.cbioportal.org/study/summary?id=prad_su2c_2019), UALCAN cancer OMICS database (http://ualcan.path.uab.edu/) to generate survival plot of TCGA-defined PCa subtypes (including ERG) and DLX1, GEPIA (http://gepia.cancer-pku.cn/) to retrieve ERG and DLX1 correlation plot in TCGA-PRAD dataset. The source data of unprocessed gel images for Figs. 2d, h, 5c, f, 6b, g, k, 7d, f and Supplementary Figs. 1c, f, 3c, d, e, 5f, g, 6b, g is provided as Source Data file.

Field-specific reporting

Please select the one below that is the best fit for your research. If you are not sure, read the appropriate sections before making your selection.

- [x] Life sciences
- [ ] Behavioural & social sciences
- [ ] Ecological, evolutionary & environmental sciences

For a reference copy of the document with all sections, see nature.com/documents/nr-reporting-summary-flat.pdf

Life sciences study design

All studies must disclose on these points even when the disclosure is negative.

Sample size	In vitro experiments were performed with 3 technical replicates every time the experiment is done and has been repeated multiple times or otherwise mentioned in the respective figure legends, main text or methods section. Any statistical constraints were not considered to choose sample size. However, we have used similar experimental conditions across biological replicates multiple time, which provided statistically significant difference between these groups. Thus, no sample-size was decided initially.
Data exclusions	No data were excluded from the analysis.
Replication	Majority of the experiments were repeated multiple times with the independent biological samples using similar experimental conditions or otherwise mentioned in the respective figure legends, main text or methods section.
Randomization	For in vivo xenograft experiment, 5-6 week old NOD/SCID mice were randomized prior to subcutaneous implantation of 22RV1-DLX1-KO and control SCR cells.<br>For mice xenograft experiment where the effect of drug treatment (BET inhibitor alone or in combination with Enzalutamide) was performed, tumors were generated by implanting 22RV1 cells in athymic nude mice, and were randomized into four treatment groups (n=6 each) once the average tumor volume reached 75mm3, namely, vehicle control, Enza (20mg/kg body weight), JQ1 (50mg/kg body weight) and a combination of Enza and JQ1.<br>For in vitro drug treatment experiments, cells were plated in 6 or 12 well culture dishes 24 hrs, and culture dish wells were randomly assigned in control or different treatment groups before the starting the drug(s) treatment.
Blinding	For scoring the immunohistochemistry staining and RNA-ISH in prostate cancer patients' tissue microarrays and mice xenografts tumors, blinding or decoding of specimens was performed. For the immunohistochemistry quantification experiment investigator was blinded during evaluation and collection of the data. For other quantification experiments such as tumor sphere assays or number of cells in foci, migration or proliferation assays, experimental conditions were blinded during data analysis. The groups were well defined during the experimental treatment, investigator could not be blinded during data collection for these quantification experiments.

Reporting for specific materials, systems and methods

We require information from authors about some types of materials, experimental systems and methods used in many studies. Here, indicate whether each material, system or method listed is relevant to your study. If you are not sure if a list item applies to your research, read the appropriate section before selecting a response.

Materials & experimental systems

n/a	Involved in the study
	[x] Antibodies
	[x] Eukaryotic cell lines
[x]	Palaeontology and archaeology
	[x] Animals and other organisms
	[x] Human research participants
[x]	Clinical data
[x]	Dual use research of concern

Methods

n/a	Involved in the study
[x]	ChIP-seq
	[x] Flow cytometry
[x]	MRI-based neuroimaging

Antibodies

Antibodies used

Following are the antibodies used in this study:
1. DLX1 (Thermo-fisher Scientific, PA5-28899)
2. E-cadherin (Cell Signaling Technology, 3195S)
3. Phospho-Akt (Cell Signaling Technology, 13038)
4. Total-Akt (Cell Signaling Technology, 9272)
5. Caspase-3 (Cell Signaling Technology,9662)
6. Cleaved PARP (Cell Signaling Technology,9541)
7. Bcl-xL (Cell Signaling Technology, 2764)
8. FoxA1/HNF3α (Cell Signaling Technology, 58613)
9. AR (Cell Signaling Technology, 5153)
10. PSA/KLK3 (Cell Signaling Technology, 5877)
11. Ki-67 (Cell Signaling Technology, 9449S)
12. Ki-67 (Agilent, IR626)
13. ALDH1A1 Cell Signaling Technology, 54135)
14. Rpb1 CTD (Cell Signaling Technology, 2629)
15. H3K9Ac Cell Signaling Technology, 9649)
16. VIM (Abcam, ab92547)
17. ERG (Abcam, ab92513)
18. β-actin (Abcam, ab6276)
19. CD44-PE, human (Miltenyi Biotec, 130-113-904)
20. CD338 (ABCG2)-PE (Miltenyi Biotec, 130-105-010)
21. Secondary HRP-conjugated anti-mouse (Jackson Immunoresearch Laboratories, 115-035-003)
22. Secondary HRP-conjugated anti-rabbit (Jackson Immunoresearch Laboratories, 111-035-144)
23. Rabbit IgG (Invitrogen, 10500C)
24. Mouse IgG (Invitrogen, 10400C)

Validation

For each primary antibody, the validation statement has been taken from the manufacturer's website or datasheet and detailed as follows:
1. Immunogen: Recombinant fragment corresponding to a region within amino acids 1 and 255 of Human Dlx1, Applications: Immunohistochemistry (Frozen) (IHC (F)) and Western Blot (WB)
2. E-Cadherin (24E10) Rabbit mAb detects endogenous levels of total E-cadherin protein; Applications: WB, Immunofluorescence (IF)
3. Phospho-Akt (Thr308) (D25E6) XP® Rabbit mAb recognizes endogenous levels of Akt1 protein only when phosphorylated at Thr308; Applications: WB, IF
4. Akt Antibody detects endogenous levels of total Akt1, Akt2 and Akt3 proteins; Applications: WB, IF
5. Caspase-3 Antibody detects endogenous levels of full length caspase-3 (35 kDa) and the large fragment of caspase-3 resulting from cleavage (17 kDa), Application: WB, Immunoprecipitation (IP), IHC
6. Cleaved PARP (Asp214) Antibody (Human Specific) detects endogenous levels of the large fragment (89 kDa) of human PARP1 produced by caspase cleavage. The antibody does not recognize full length PARP1 or other PARP isoforms, Application: WB.
7. Bcl-xL (54H6) Rabbit mAb detects endogenous levels of total Bcl-xL protein. The antibody does not cross-react with other Bcl-2 family members, Applications: WB, IP, IHC, IF and flow cytometry (FC).
8. FoxA1/HNF3α (D7P9B) Rabbit mAb recognizes endogenous levels of total FoxA1/HNF3α protein, Applications: WB, Chromatin immunoprecipitation (ChIP).
9. Androgen Receptor (D6F11) XP® Rabbit mAb detects endogenous levels of total androgen receptor protein; Applications: WB, IHC, IF, ChIP .
10. PSA/KLK3 (D2A8) Rabbit mAb recognizes endogenous levels of total PSA/KLK3 protein; Applications: WB, IF
11. Ki-67 (8D5) Mouse mAb recognizes endogenous levels of total Ki-67 protein, Applications: IHC, IF, FC.
12. Ki-67 (IR626) Mouse mAb (MIB-1), Immunogen: Human recombinant peptide corresponding to a 1002 bp Ki-67 cDNA fragment; Applications: Formalin, HIER
13. ALDH1A1 (54135) Rabbit mAb recognizes endogenous levels of ALDH1A1; Application: WB, IP, IHC, F
14. Rpb1 CTD (4H8) Antibody detects endogenous levels of total Rpb1 protein (both phosphorylated and unphosphorylated forms); Applications: WB, ChIP
15. Acetyl-Histone H3 (Lys9) (C5B11) Rabbit mAb detects endogenous levels of histone H3 only when acetylated on Lys9; Applications: WB, ChIP

16. Immunogen: Synthetic peptide within Human Vimentin aa. 400 to the C-terminus (C terminal) (acetyl). The exact sequence is proprietaryApplications: ICC/IF, WB, Flow Cyt, IHC-P
17. Immunogen: Synthetic peptide within Human ERG aa 450 to the C-terminus. Applications: WB, IF
18. Immunogen: Synthetic peptide corresponding to beta Actin aa 1-14 (N terminal) conjugated to Keyhole Limpet Haemocyanin (KLH). Sequence: DDDIAALVIDNGSGK; Applications: WB
19. Immunogen: CD44, also known as CD44s, EMCR III, H-CAM, Pgp-1, Applications: FC, MACSima Imaging Cyclic Staining (MICS)
20. Immunogen: CD338 (ABCG2), Alternative names of antigen ABCG2, Breast cancer resistance protein (BCRP, BCRP1), CDw338, Mitoxantrone resistance-associated protein (MXR), Placenta-specific ATP-binding cassette transporter, Urate exporter, Application: FC

Eukaryotic cell lines

Policy information about cell lines

Cell line source(s)	Prostate cancer cell lines (22RV1, VCaP, LNCaP and PC3) and benign prostate epithelial cells (RWPE1) were obtained from the American Type Culture Collection (ATCC) and were cultured as per the ATCC recommended guidelines. HEK293FT cells were obtained from Thermo Fisher Scientific and were cultured in the recommended conditions. 42-D ENZR cell line was generated using LNCaP cell line and was kindly gifted by Dr. Amina Zoubeidi (PMID: 27784708), University of British Columbia, Canada. C4-2 shAR cell line was generated and kindly gifted by Paul Rennie, University of British Columbia, Vancouver. To generate these cells, C4-2 cell line was obtained from ATCC and stable knockdown of androgen receptor (AR) was performed using short hairpin based RNA (shRNA) system (PMID: 17079486).
Authentication	Cell line authentication was done using short tandem repeat (STR) profiling at the Lifecode Technologies Private Limited, Bangalore, and DNA Forensics Laboratory, New Delhi.
Mycoplasma contamination	Mycoplasma contamination test was routinely carried out for all the cell lines using PlasmoTest mycoplasma detection kit (InvivoGen). All cell lines tested negative for mycoplama contamination.
Commonly misidentified lines (See ICLAC register)	No misidentified cell lines were used in this study.

Animals and other organisms

Policy information about studies involving animals; ARRIVE guidelines recommended for reporting animal research

Laboratory animals	NOD.CB17-Prkdcscid/J (NOD/SCID) immunodeficient male mice obtained from Jackson Laboratory and NU(NCr) and Foxn1nu athymic nude male mice procured from Hylasco Biotechnology Pvt. Ltd., India (authorized distributor for Charles River Research Models) were used in the study. Mice colonies were maintained in specific-pathogen-free (SPF) facility as per the guidelines. 5-6 week old male mice were used for all the xenograft studies.
Wild animals	No wild animals were used in this study.
Field-collected samples	No field collected samples were used.
Ethics oversight	All mice experiment procedures were approved by the Committee for the Purpose of Control and Supervision of Experiments on Animals (CPCSEA) and abide to all regulatory standards of the Institutional Animal Ethics Committee of the Indian Institute of Technology Kanpur.

Note that full information on the approval of the study protocol must also be provided in the manuscript.

Human research participants

Policy information about studies involving human research participants

Population characteristics	The human research participants included in the study were males suffering from prostate cancer (PCa). Prostate Cancer tissue microarrays (TMA) obtained from Henry Ford Health System (HFHS), Detroit, Michigan, USA comprises of PCa biospecimens collected after radical prostatectomy of patients belonging to the age group ranging from ~44-76 years. All patients were suffering with localized PCa, with few showing lymph node metastasis and were not administered with any hormone therapy, except for three cases.<br>The TMA containing biospecimens of metastatic castrate-resistant prostate cancer (mCRPC) was obtained from University of Washington, and was comprising of patients with age group of ~45-90 years at the time of autopsy. All patients were treated with anti-androgen drugs, except for one.<br>All the metastatic tissues from distant organ sites were obtained from patients who died of mCRPC within 8 hours after their death. Visceral metastases were identified at the gross level, bone biopsies were obtained from ~16-20 different sites and metastases was identified at histological level.
Recruitment	The PCa biospecimens included in the HFHS TMA were obtained from the patients who underwent radical proctectomy at the Henry Ford Health System (HFHS), Detroit, Michigan, USA, other than that there was no prior selection bias.<br>The mCRPC patients were consented to a rapid autopsy within the catchment area of the University of Washington Medical Center, influencing the diversity and socio-economic status of the patient cohort.
Ethics oversight	Institutional Review Board approvals from the Henry Ford Health System (IRB#10375) and University of Washington

(IRB#2341) and informed consents were received from all the participants prior to inclusion in the study. All patients' specimens used in this study were collected in accordance with the Declaration of Helsinki.

Note that full information on the approval of the study protocol must also be provided in the manuscript.

Flow Cytometry

Plots

Confirm that:

- [x] The axis labels state the marker and fluorochrome used (e.g. CD4-FITC).
- [x] The axis scales are clearly visible. Include numbers along axes only for bottom left plot of group (a 'group' is an analysis of identical markers).
- [x] All plots are contour plots with outliers or pseudocolor plots.
- [x] A numerical value for number of cells or percentage (with statistics) is provided.

Methodology

Sample preparation	For cell cycle distribution, cells were trypsinized and fixed in 70% ethanol followed by staining with propidium iodide (PI) (50µg/ml) (BioLegend, Cat # 421301) for 20 minutes as per the manufacture's protocol.<br>For apoptotic studies, cells were dissociated using StemPro™ Accutase™ (ThermoFisher Scientific) and were washed with cold 1X PBS and resuspended in 1X binding buffer (1x106 cells/ml). Subsequently, 1x105 cells were stained using PE Annexin V Apoptosis Detection Kit I (BD Biosciences, Cat # 559763) following to the manufacture's protocol.<br>Aldefluor assay was performed to determine aldehyde dehydrogenase (ALDH) enzymatic activity using Aldefluor kit (Stem Cell Technologies, Catalog #01700) following manufacturer's guidelines. Briefly, cells were trypsinized and washed with 1X PBS followed by resuspension in 1ml of Aldefluor assay buffer. Activated Aldefluor substrate (5µL) was added to the cells and were divided in two conditions, with and without ALDH inhibitor, diethylaminobenzaldehyde (DEAB). After 30 minutes of incubation at 37°C, cells were centrifuged and resuspended in 500µL of Aldefluor assay buffer.<br>For stem cell markers, cells were stained with PE/Cy7 anti-human CD44 antibody (Miltenyi Biotec, 130-113-904, 1:50) and CD338-PE (ABCG2-PE, Miltenyi Biotec, 130-105-010, 1:40) followed by 1-hour incubation at 4°C.
Instrument	Beckman Coulter's CytoFLEX platform or BD FACSMelody™ Cell Sorter was used
Software	Data was analyzed with FlowJo software v10.6.1.
Cell population abundance	Flow cytometry experiments were performed on pure populations of isogeneic cell lines. No cell sorting was done.
Gating strategy	For cell cycle distribution, ~10000 events were acquired and analyzed using in-built univariate model of FlowJo software.<br>For apoptosis assay, ~10000 events were acquired. Quadrants were gated on Annexin V (PE) versus 7AAD (PerCP) channel dot plots using unstained, Annexin V (PE) and 7AAD (PerCP) single stained cells as controls. The quadrants were defined as lower left quadrant Annexin-/7AAD- (viable), Annexin+/7AAD- lower right quadrant (early apoptotic), Annexin-/7AAD+ upper left quadrant (necrotic), Annexin+7AAD+ upper right quadrant (late apoptotic) cells.<br>The ALDH activity was detected in FITC channel. ~100000 events were acquired and gate was applied to identify viable-cell population using forward scatter (FSC) and side scatter (SSC) dot plot. Next, dot plot was generated with FITC channel versus SSC and gate on the control population was applied using DEAB treated samples. The same gate was applied over corresponding samples without DEAB to identify ALDH-positive population.<br>For cell-surface markers ~100000 events were acquired, gate was applied to identify viable-cell population using forward scatter (FSC) and side scatter (SSC) dot plot. Histograms were generated for antibody-stained samples and were compared to their isotype controls.

- [x] Tick this box to confirm that a figure exemplifying the gating strategy is provided in the Supplementary Information.
