# Supplementary material for: Transcriptional network involving ERG and AR orchestrates Distal-less homeobox-1 mediated prostate cancer progression
Source: Nat Commun. 2021 Sep 7;12:5325. doi: 10.1038/s41467-021-25623-2 (PMC8423767; doi:10.1038/s41467-021-25623-2)

Figure 2b

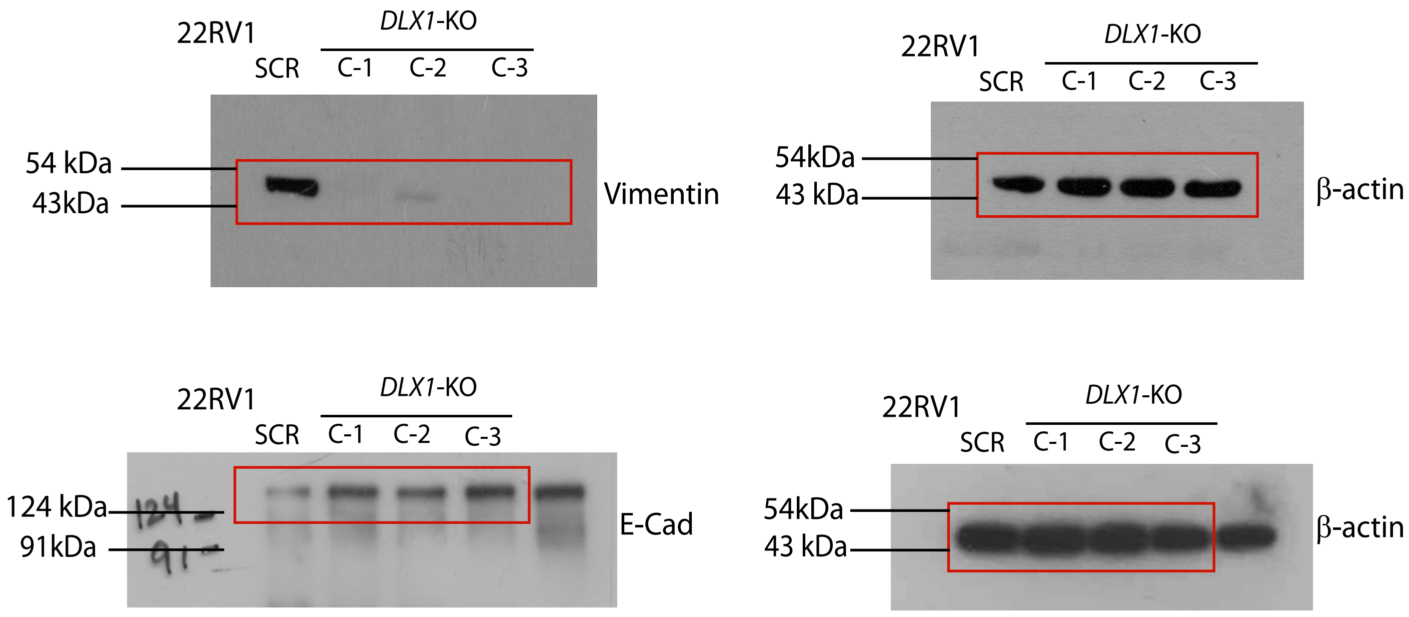
22RV1
DLX1-KO
SCR
C-1
C-2
C-3
54 kDa
43kDa
Vimentin
22RV1
DLX1-KO
SCR
C-1
C-2
C-3
54kDa
43 kDa
β-actin
22RV1
DLX1-KO
SCR
C-1
C-2
C-3
124 kDa
91kDa
E-Cad
22RV1
DLX1-KO
SCR
C-1
C-2
C-3
54kDa
43 kDa
β-actin

Figure 2c

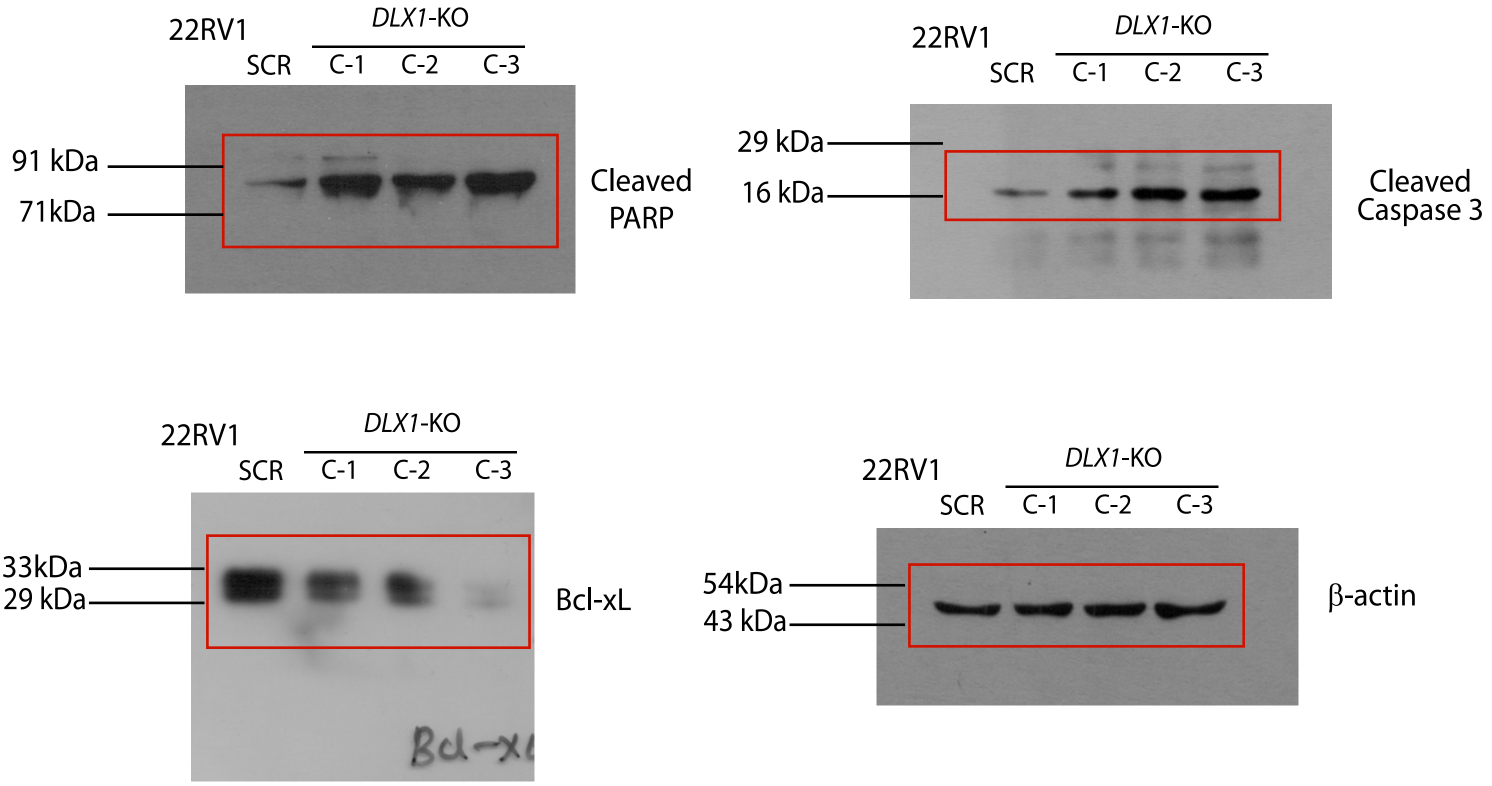
22RV1
DLX1-KO
SCR
C-1
C-2
C-3
91 kDa
71kDa
Cleaved PARP
29 kDa
16 kDa
Cleaved Caspase 3
33kDa
29 kDa
Bcl-xL
54kDa
43 kDa
β-actin

Figure 5c

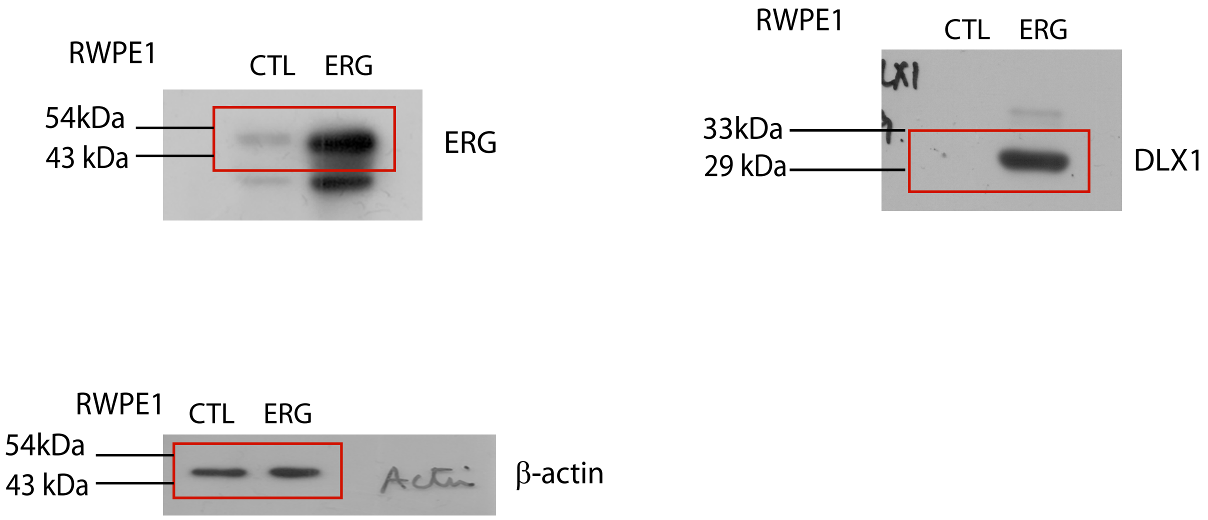
RWPE1
CTL
ERG
54kDa
43 kDa
ERG
RWPE1
CTL
ERG
33kDa
29 kDa
DLX1
RWPE1
CTL
ERG
54kDa
43 kDa
β-actin

Figure 5f

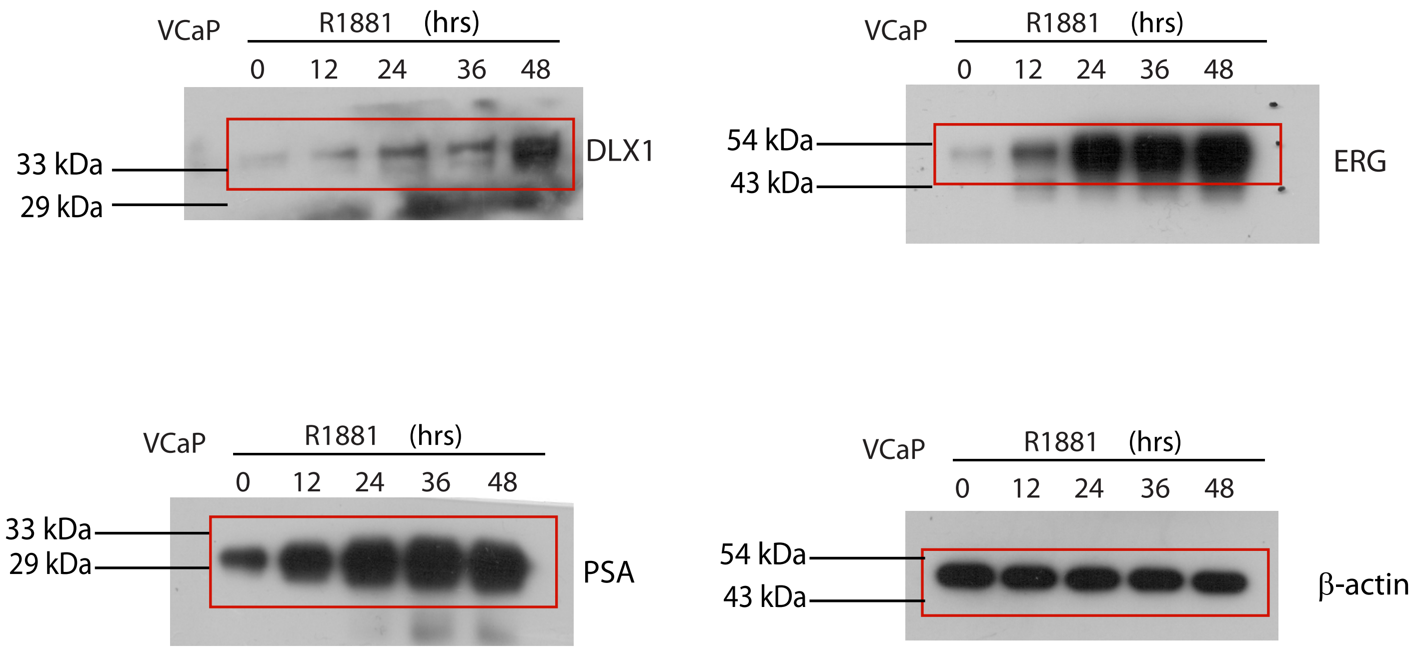

VCaP
R1881 (hrs)
0 12 24 36 48
33 kDa
29 kDa
DLX1
VCaP
R1881 (hrs)
0 12 24 36 48
54 kDa
43 kDa
ERG
VCaP
R1881 (hrs)
0 12 24 36 48
33 kDa
29 kDa
PSA
VCaP
R1881 (hrs)
0 12 24 36 48
54 kDa
43 kDa
β-actin

Figure 6b

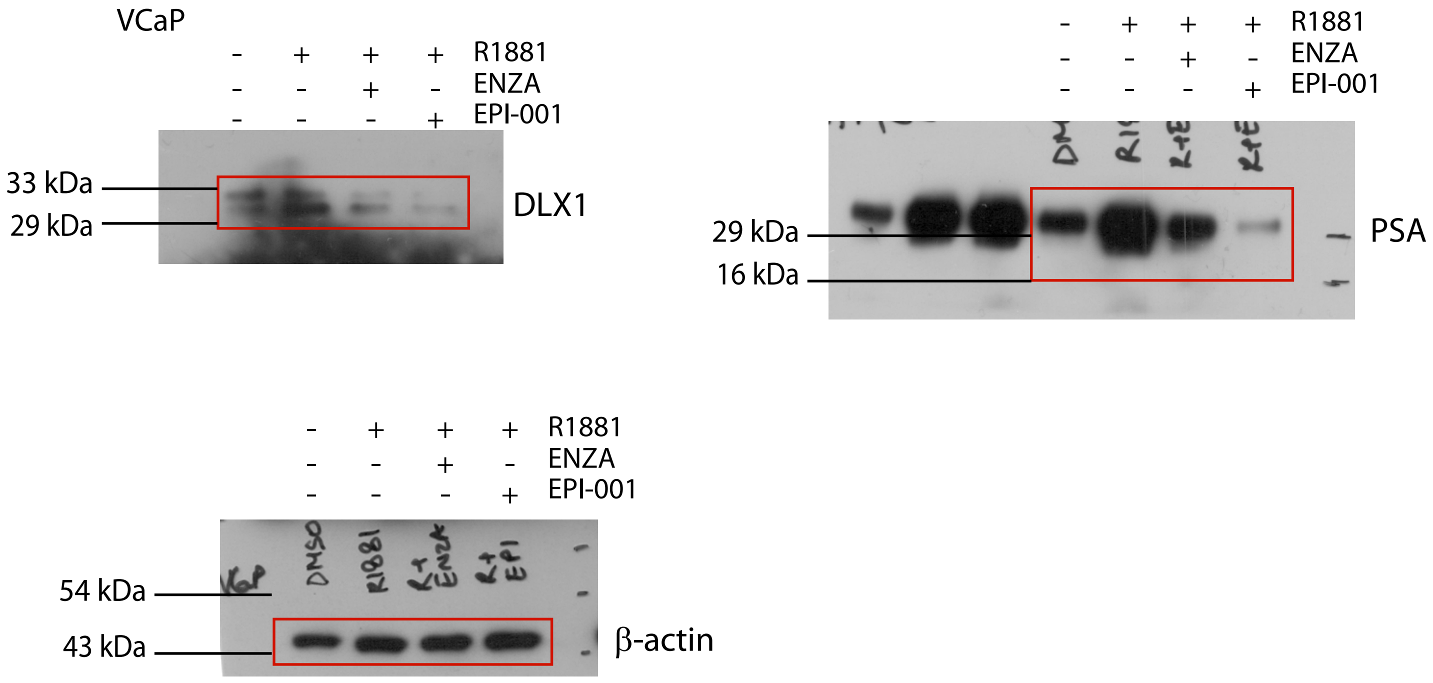

VCaP
- + + + R1881
- - + - ENZA
- - - + EPI-001
33 kDa
29 kDa
DLX1
- + + + R1881
- - + - ENZA
- - - + EPI-001
29 kDa
16 kDa
PSA
- + + + R1881
- - + - ENZA
- - - + EPI-001
54 kDa
43 kDa
β-actin

Figure 6h

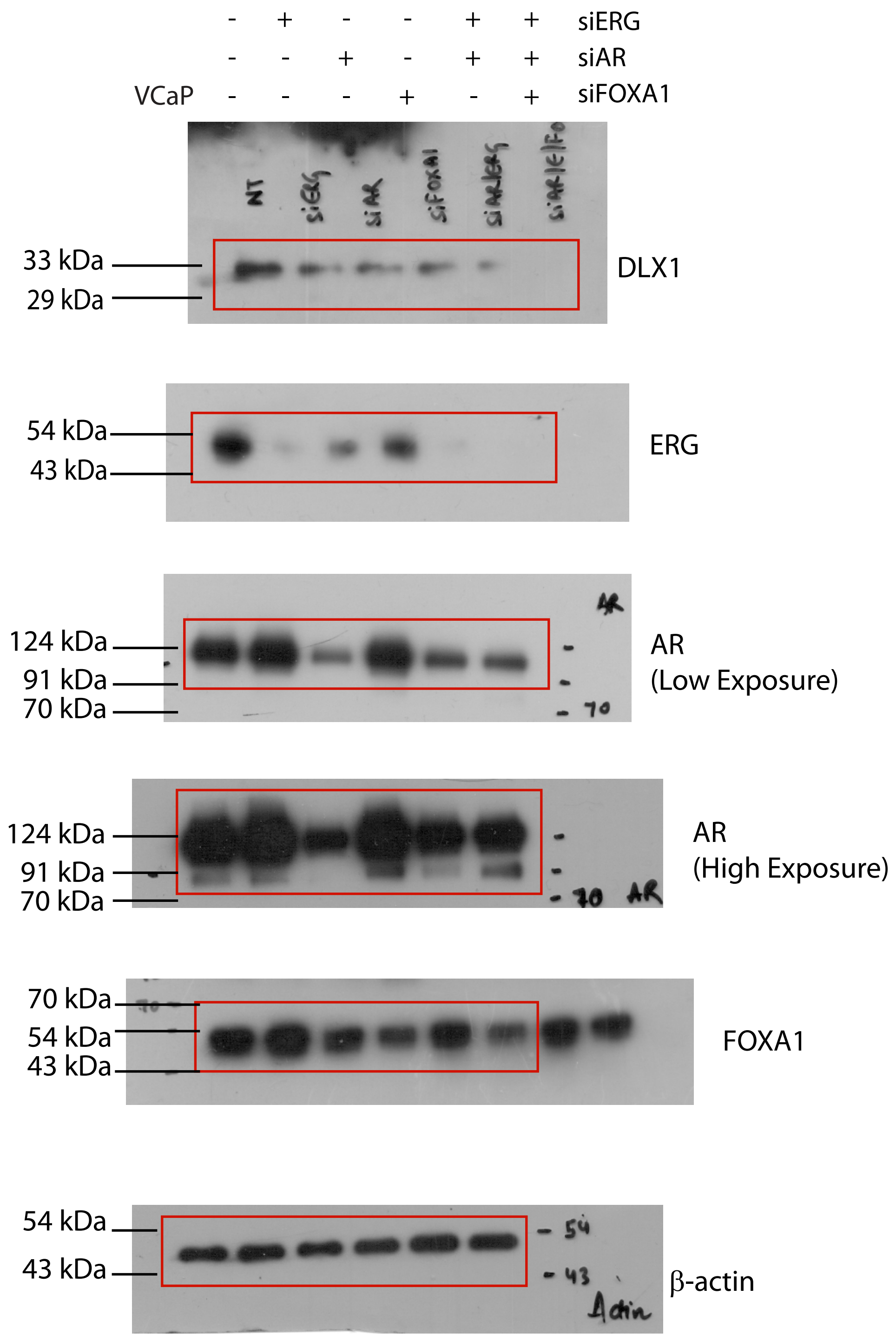

- + - - + + siERG
- - + - + + siAR
VCaP - - - + - + siFOXA1
33 kDa
29 kDa
DLX1
54 kDa
43 kDa
ERG
124 kDa
91 kDa
70 kDa
AR
(Low Exposure)
124 kDa
91 kDa
70 kDa
AR
(High Exposure)
70 kDa
54 kDa
43 kDa
FOXA1
54 kDa
43 kDa
β-actin

Figure 6l

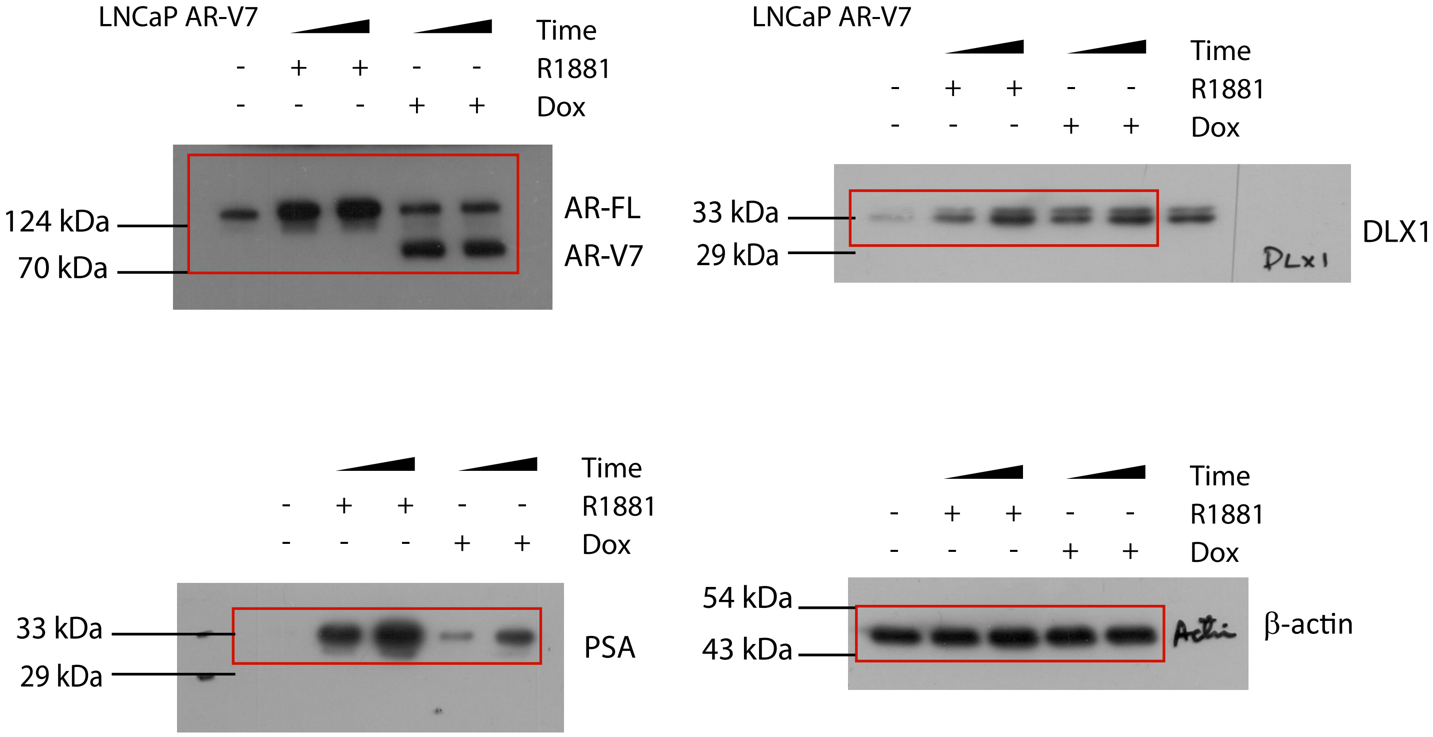

LNCaP AR-V7
Time
R1881
Dox
- + + - -
- - - + +
124 kDa
70 kDa
AR-FL
AR-V7
LNCaP AR-V7
Time
R1881
Dox
33 kDa
29 kDa
DLX1
Time
R1881
Dox
33 kDa
29 kDa
PSA
Time
R1881
Dox
54 kDa
43 kDa
β-actin

Figure 7d

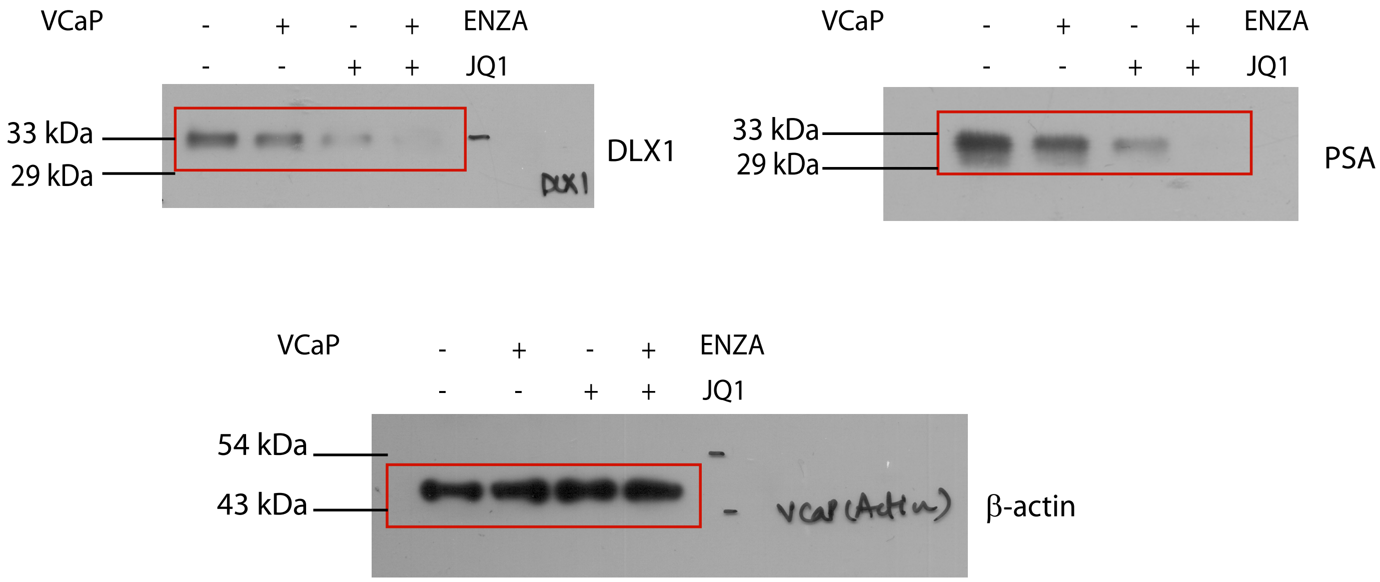
VCaP
- + - + ENZA
- - + + JQ1
33 kDa
29 kDa
DLX1
VCaP
- + - + ENZA
- - + + JQ1
33 kDa
29 kDa
PSA
VCaP
- + - + ENZA
- - + + JQ1
54 kDa
43 kDa
β-actin

Figure 7f

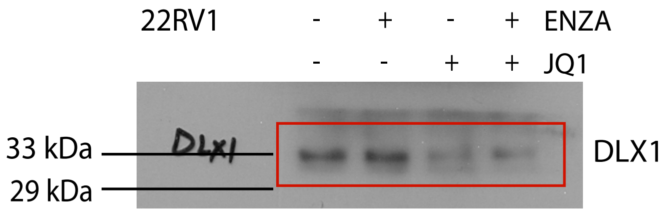
22RV1
- + - + ENZA
- - + + JQ1
33 kDa
29 kDa
DLX1
DLX1

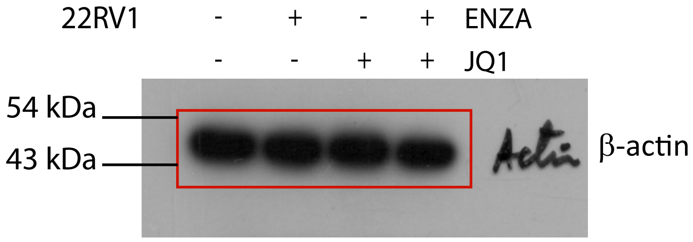
22RV1
- + - + ENZA
- - + + JQ1
54 kDa
43 kDa
Actin
β-actin

Supplementary Figure 1c

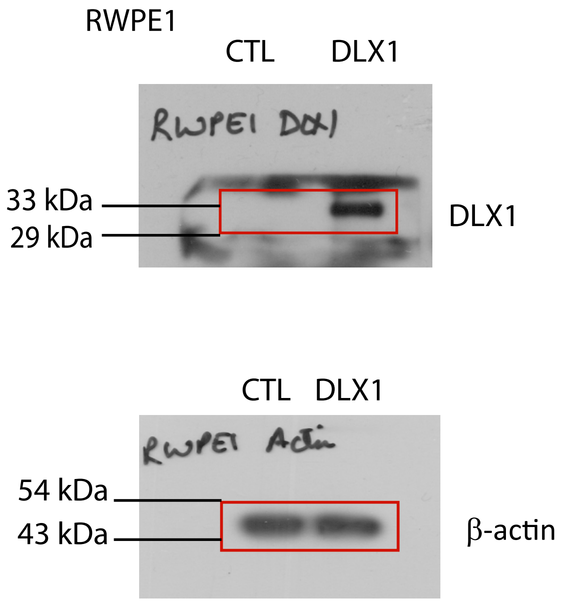

Supplementary Figure 1f

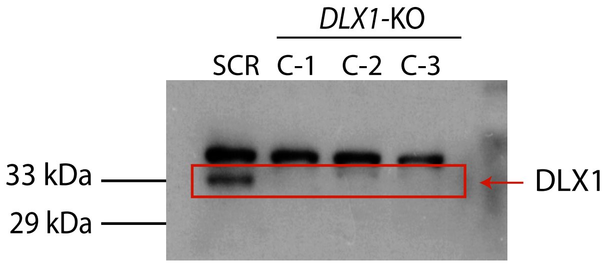

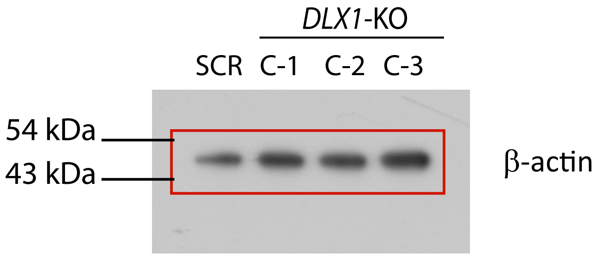

Supplementary Figure 3a

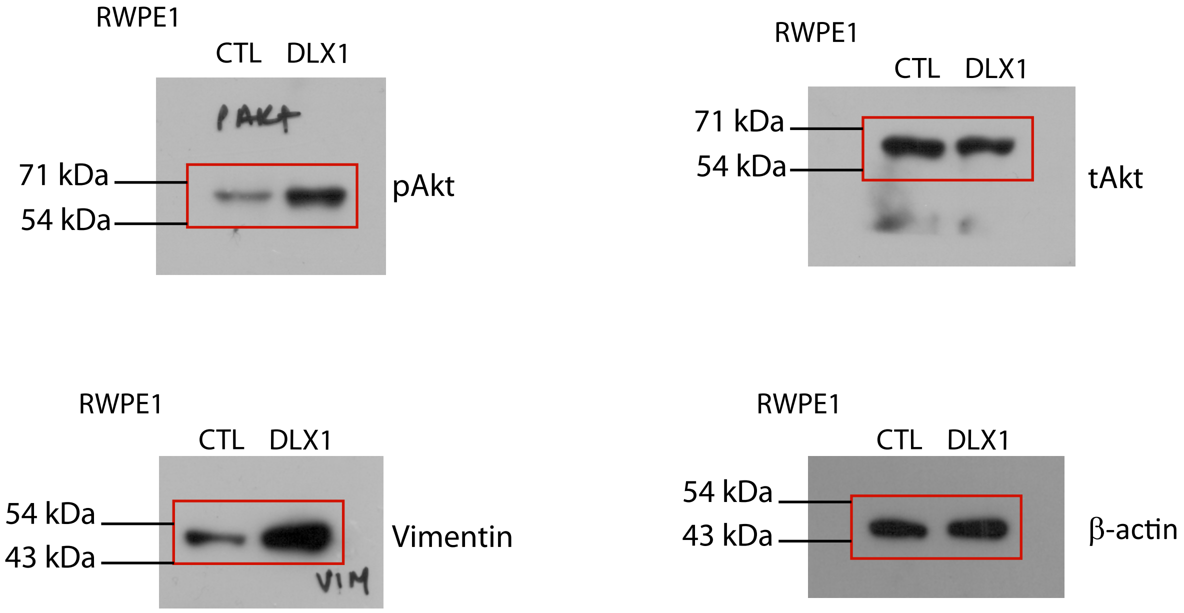

Supplementary Figure 3b

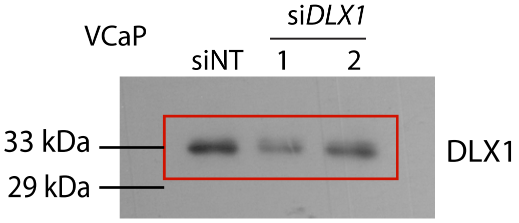

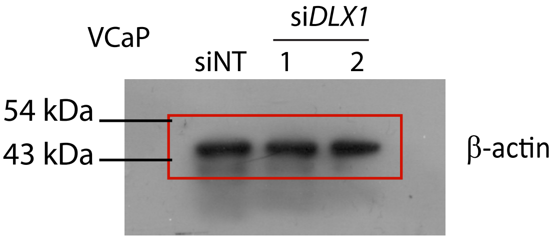

Supplementary Figure 3d

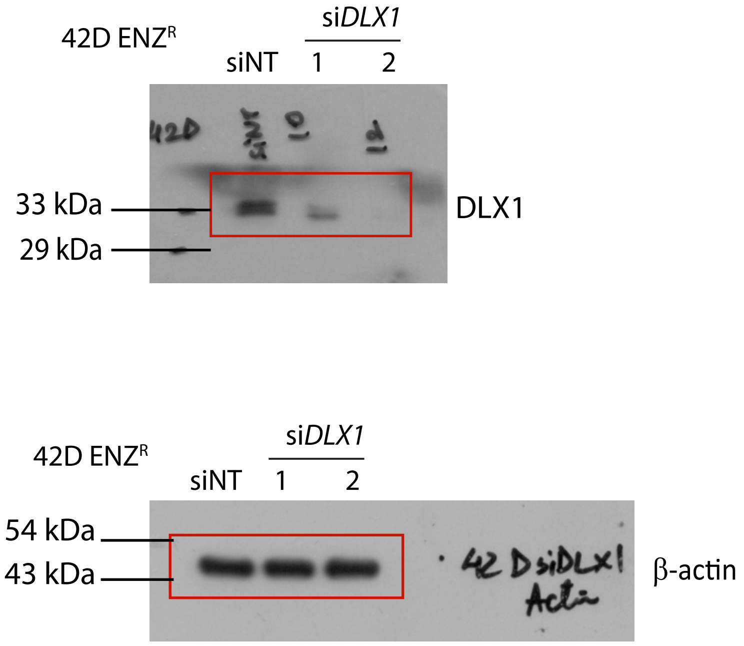

Supplementary Figure 5e

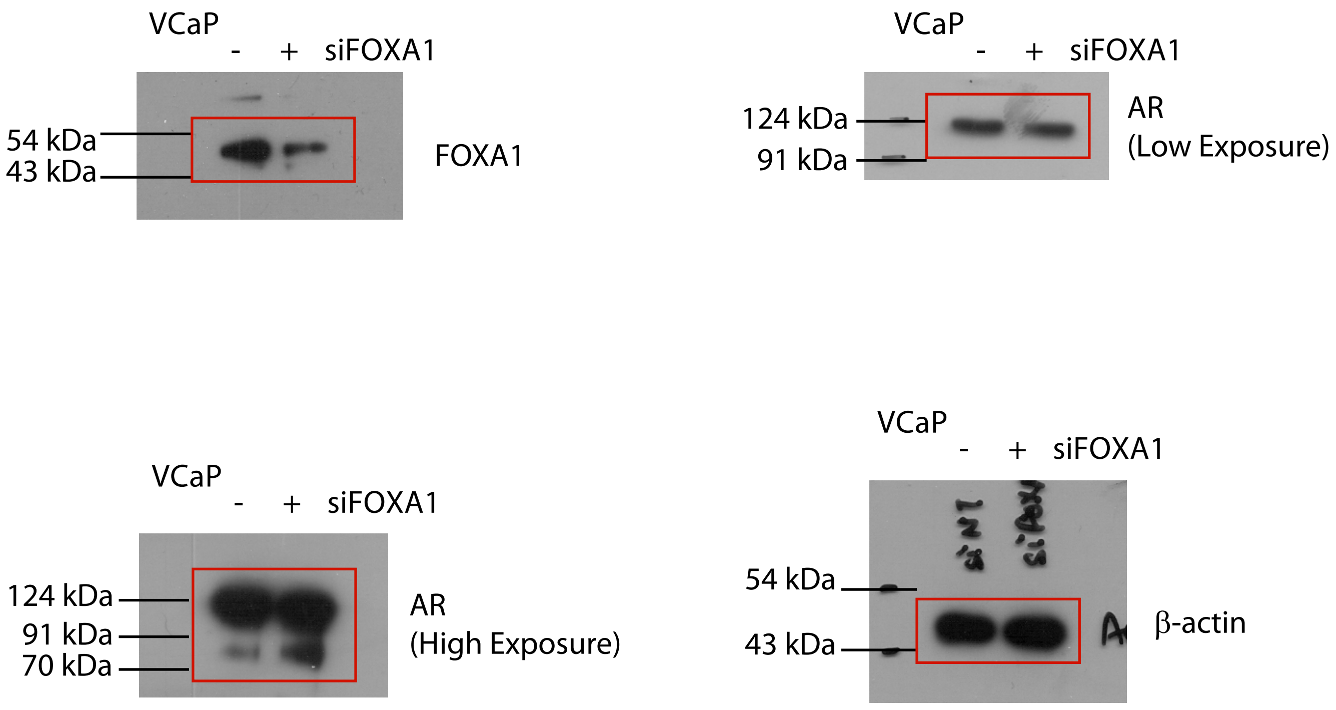

Supplementary Figure 5f

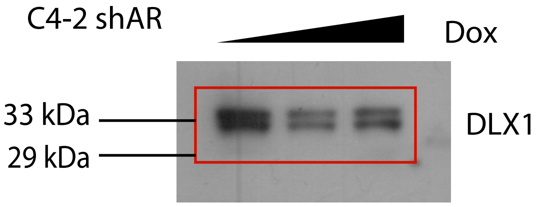

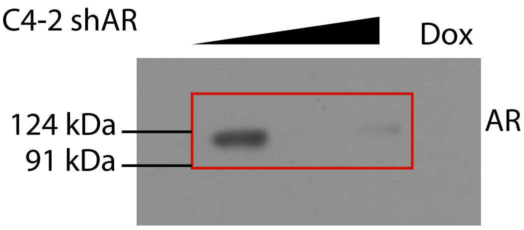

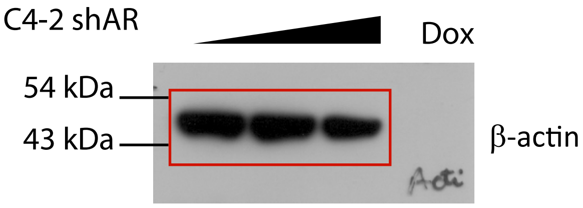

Supplementary Figure 6b

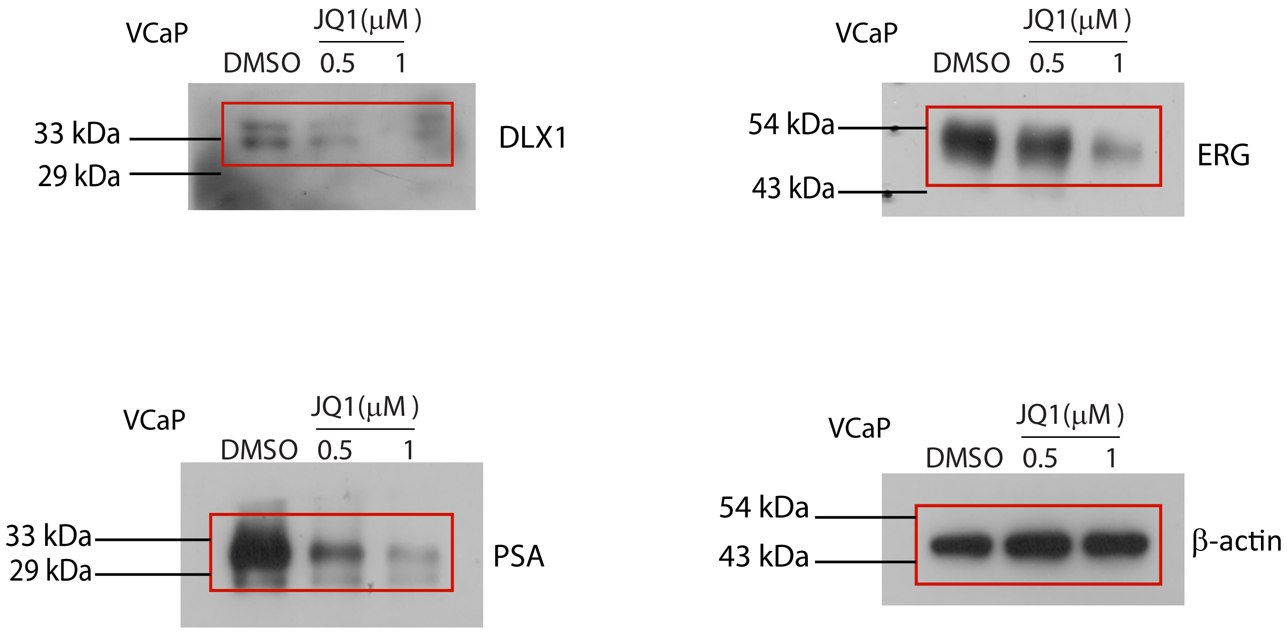

Supplementary Figure 6h

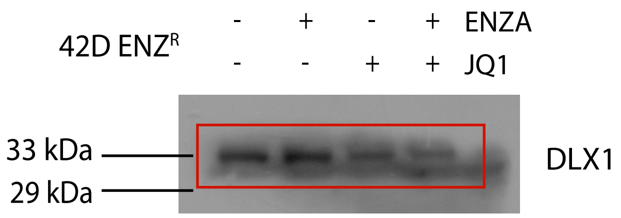

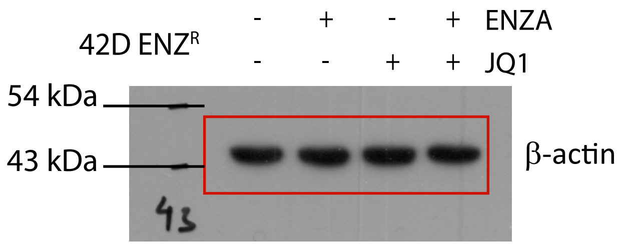

Supplement: Supplementary file 6 — Source Data [file 41467_2021_25623_MOESM6_ESM.zip › Unprocessed Gels Source Data.pdf]
